# The cingulum: a central hotspot for the battle against chronic intractable pain?

**DOI:** 10.1093/braincomms/fcae368

**Published:** 2024-10-16

**Authors:** Linda Kollenburg, Hisse Arnts, Alexander Green, Ido Strauss, Kris Vissers, Saman Vinke, Erkan Kurt

**Affiliations:** Radboud University Medical Center, Department of Neurosurgery, Functional Neurosurgery Unit, Nijmegen, 6525 GA, Netherlands; Radboud University Medical Center, Department of Neurosurgery, Functional Neurosurgery Unit, Nijmegen, 6525 GA, Netherlands; Oxford Functional Neurosurgery and Experimental Neurology Group, Nuffield Department of Clinical Neuroscience and Surgery, University of Oxford, Oxford OX39DU, UK; Tel Aviv Medical Center, Department of Neurosurgery, Functional Neurosurgery Unit, Tel Aviv 6801298, Israel; Radboud University Medical Center, Department of Pain and Palliative Care, Nijmegen, 6525 GA, Netherlands; Radboud University Medical Center, Department of Neurosurgery, Functional Neurosurgery Unit, Nijmegen, 6525 GA, Netherlands; Radboud University Medical Center, Department of Neurosurgery, Functional Neurosurgery Unit, Nijmegen, 6525 GA, Netherlands; Radboud University Medical Center, Department of Pain and Palliative Care, Nijmegen, 6525 GA, Netherlands

**Keywords:** cingulum, cingulate cortex, cingulotomy, chronic pain, deep brain stimulation

## Abstract

Chronic pain causes a major burden on patient’s lives, in part due to its profound socioeconomic impact. Despite the development of various pharmacological approaches and (minor) invasive treatments, a subset of patients remain refractory, hence why alternative targeted neurosurgical interventions like cingulotomy and deep brain stimulation of the anterior cingulate cortex should be considered in the last resort. Despite clinical evidence supporting the potential of these treatments in the management of chronic intractable pain, physicians remain reluctant on its clinical implementation. This can be partially attributed to the lack of clear overviews summarizing existent data. Hence, this article aims to evaluate the current status of cingulotomy and deep brain stimulation of the anterior cingulate cortex in the treatment of chronic intractable pain, to provide insight in whether these neurosurgical approaches and its target should be reconsidered in the current era. In the current study, a literature searches was performed using the PubMed database. Additional articles were searched manually through reviews or references cited within the articles. After exclusion, 24 and 5 articles remained included in the analysis of cingulotomy and deep brain stimulation of the anterior cingulate cortex, respectively. Results indicate that various surgical techniques have been described for cingulotomy and deep brain stimulation of the anterior cingulate cortex. Cingulotomy is shown to be effective 51–53% and 43–64% of patients with neoplastic and non-neoplastic pain at ≤6 months follow-up, and 82% (9/11) and 76% (90/118) at ≥ 12months follow-up, respectively. With regard to deep brain stimulation of the anterior cingulate cortex, no data on neoplastic pain was reported, however, 59% (10/17) and 57% (8/14) of patients with non-neoplastic pain were considered responders at ≤ 6 months and ≥ 12months follow-up, respectively. The most reported adverse events include change in affect (>6.9%, >29/420) and confusion (>4.8%, >20/420) for cingulotomy, and infection (12.8%, 6/47), seizures (8.5%, 4/47) and decline in semantic fluency (6.4%, 3/47) for deep brain stimulation of the anterior cingulate cortex. It can be concluded that cingulotomy and deep brain stimulation of the anterior cingulate cortex are effective last resort strategies for patients with refractory non-neoplastic and neoplastic pain, especially in case of an affective emotional component. Future research should be performed on the cingulum as a neurosurgical target as it allows for further exploration of promising treatment options for chronic intractable pain.

## Graphical Abstract

**Graphical Abstract:**
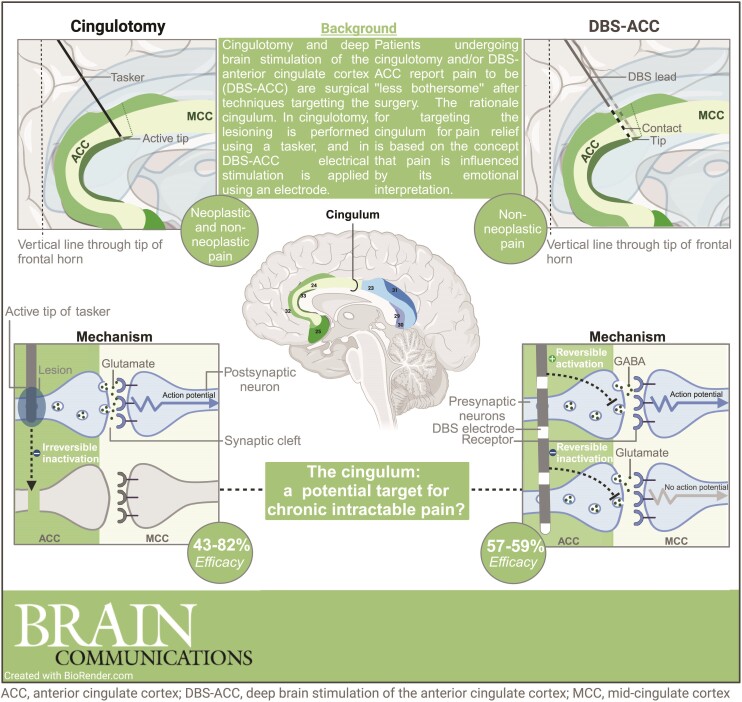

## Introduction

Chronic pain can be considered a silent epidemic, affecting one in five adults of the general population.^1^ It manifests as a consistent sensation of discomfort with descriptions varying from aching, dull, and throbbing to sharp, stabbing, shooting and/or burning forms of pain.^2,3^ Chronic pain is associated with a high cost and burden to healthcare systems, causing significant disabilities affecting daily life, as well as social isolation, and a higher risk of suicide.^4-6^ Despite the presence of various pharmacological and (minor) invasive strategies for chronic pain, a substantial proportion of patients remain treatment refractory and, therefore, are possible candidates for targeted neurosurgical interventions like cingulotomy and deep brain stimulation of the anterior cingulate cortex (DBS-ACC).^7^ Cingulotomy is a stereotactical procedure in which bilateral lesions are made in the anterior cingulate cortex (ACC), consequently leading to alterations in pain processing due to the destruction of fibre connections involved in emotional regulation. Almost half a century after the first cingulotomy was performed, deep brain stimulation (DBS) was introduced as a less-destructive alternative to treat intractable chronic pain.^8^ DBS-ACC involves implantation of one or more electrodes within the cingulum that are connected to a neurostimulator that provides the electricity to reversibly modulate ACC activity. Cingulotomy and DBS-ACC have been reported to cause substantial pain relief in patients with various chronic intractable pain disorders.^9,10^ As the ACC is most predominantly involved in emotional regulation, autonomic integration and affect related to pain, the rationale for targeting the ACC for pain relief is based on the concept that pain is influenced by its emotional interpretation.^11-13^ Patients undergoing cingulotomy and DBS-ACC describe pain to be ‘less bothersome’ after surgery.^14,15^ The potential of the cingulate cortex as a neurosurgical target for chronic pain was already suggested in the mid-20th century when cingulotomy caused pain relief in patients with various mental and chronic pain disorders.^16^ Recent evidence from both animal and human studies shows the presence of enhanced excitatory synaptic neurotransmission, dendritic dysfunction, decreased inhibition and opioid receptor binding, and altered somatosensory processing in the ACC of patients with various chronic pain disorders, thereby reboosting interests in this central ‘hub’.^17-22^

Despite historical and more recent evidence supporting use of the ACC as a neurosurgical target, cingulotomy and DBS-ACC do not belong to the standard neurosurgical arsenal for chronic intractable pain. Heterogeneity in pain syndromes, unstandardized surgical techniques, but also the limited availability of clinical reports and absence of clear overviews summarizing existent data, likely prohibit further clinical implementation of these neurosurgical techniques for patients with chronic pain. Therefore, the open questions on the efficacy of cingulotomy and DBS-ACC for refractory pain remain a matter of ongoing debate. Hence, this article aims to evaluate the current status of cingulotomy and DBS-ACC for non-neoplastic and neoplastic pain, to provide insight in whether these neurosurgical approaches and its target should be reconsidered in the current era.

## Materials and methods

### Literature search

**Figure 1:**
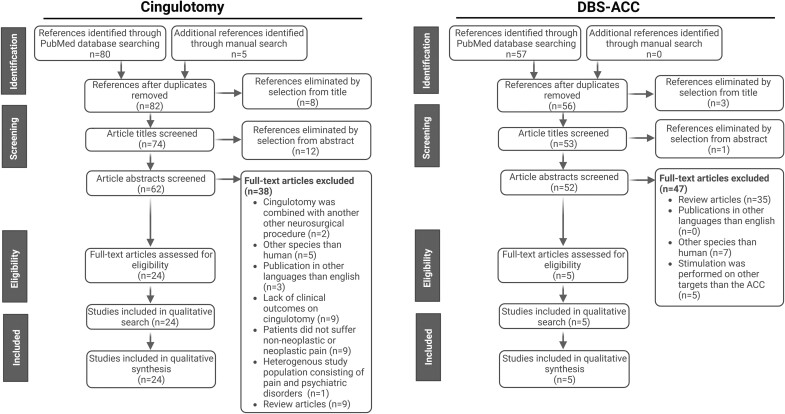
Literature search overview of the literature search for cingulotomy and DBS-ACC.

In the current systematic review, two distinct literature searches for cingulotomy and DBS-ACC were performed using PubMed. For cingulotomy, the following search strategy was used: ((*pain[MeSH] OR pain[tiab]*) *AND* (*cingulotomy[tiab] OR cingulumotomy[tiab]*)). For DBS-ACC, the following search strategy was used: ((*pain[MeSH] OR pain[tiab]*) *AND* (*DBS[tiab] OR deep brain stimulation[tiab]*) *AND* (*cingulum[tiab] OR cingulate[tiab] OR ACC[tiab]*)). For both searches, only studies focussing on stimulation/destruction of the cingulum bundle and/or cingulate cortex to relieve pain were used in the analysis. Additional articles were hand searched through reviews or references cited within the articles. Reviews that were only used to find additional studies were not included in the current analysis. Articles lacking sufficient details on the technique and/or efficacy of cingulotomy and/or DBS-ACC were also excluded. After exclusion, 24 studies remained included in the analysis of cingulotomy and 5 for DBS-ACC (Fig. 1). Articles covering the mechanisms underlying cingulotomy and DBS-ACC were collected manually in an additional search. Created with BioRender.com.

### Literature analysis and data synthesis

The primary outcomes of this study are to evaluate the (i) mechanism, (ii) surgical technique, (iii) efficacy and (iv) safety of cingulotomy and DBS-ACC in patients with both refractory non-neoplastic and neoplastic pain. These outcomes were used to compare the efficacy of cingulotomy/DBS-ACC between patients with non-neoplastic and neoplastic pain, as well as to compare the findings on cingulotomy with outcomes obtained for DBS-ACC. Responders were defined as those having at least 30% reduction in visual analogue scale (VAS)/numeric rating scale (NRS) score and/or pain medication intake. As we anticipated that the reported outcomes would be heterogeneous (30% responder rate (30RR) or 50% responder rate (50RR) in the majority of studies, but allowing any percentage at and over 30%), we chose to report an overall responder rate (ORR), only including subjects who meet the responder definition as proposed in this study. In case no responder definition was provided by the authors, but alterations in pain intensity were described accurately, the change in VAS/NRS/pain medication intake was calculated manually, and those with more than 30% change were also defined responders. For studies using an alternative approach for quantifying pain relief, categories like ‘good’, ‘significant’ and/or ‘excellent’ were included instead for the ORR. Regarding the surgical technique, the location of the surgical targets was noted as a single value. In studies reporting a range value, the mean was taken as a point value for the calculations and visualisations.

## Results

### Cingulotomy

In 1948, cingulotomy was introduced as treatment for patients with psychiatric disorders including obsessions, depression, anxiety and schizophrenia.^23^ Several studies showed improvements in depression, as well as insomnia and anxiety in patients with comorbid (severe) depression after receiving cingulotomy.^24,25^ As chronic pain and depressive disorders are common comorbidities, cingulotomy was later also expected to be effective for patients suffering chronic pain conditions.^26,27^

#### Mechanism

**Figure 2:**
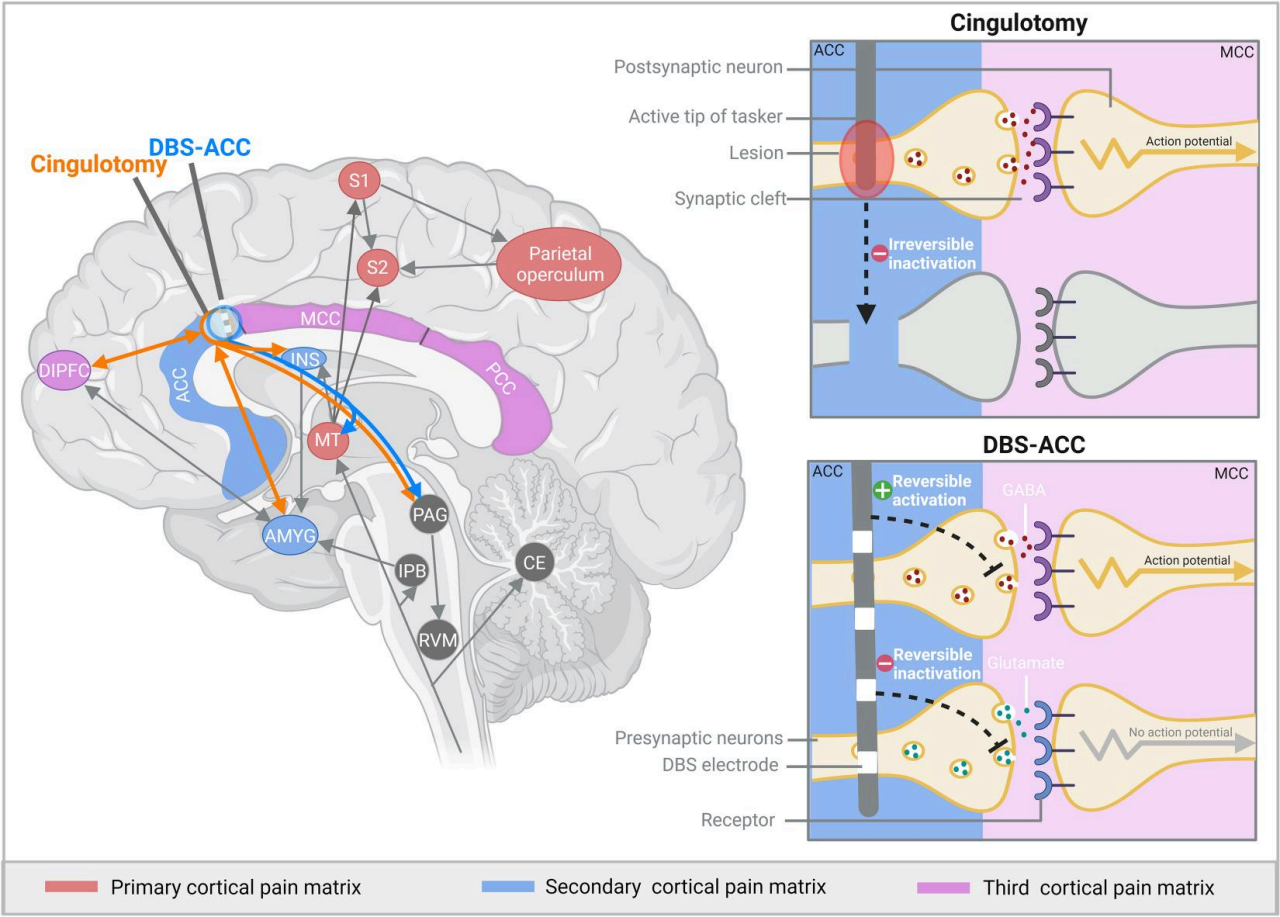
**Mechanism of cingulotomy and DBS-ACC overview of the hypothesized mechanism of action underlying cingulotomy and DBS-ACC.** AMYG, amygdala; ACC, anterior cingulate cortex; CE, cerebellum; dIPFC, dorsolateral prefrontal cortex; DBS, deep brain stimulation; INS, insular cortex; IPB, interpeduncular nucleus; PCC, posterior cingulate cortex; MCC, mid cingulate cortex; MT, middle temporal nucleus; RVM, rostral ventromedial medulla; S1, primary somatosensory cortex; S2, secondary somatosensory cortex. *Please note that the variability in angle between the electrodes inserted for the DBS-ACC and cingulotomy was necessary to allow clear visualisation of the distinct structures affected by both approaches, in reality, similar angles are used for cingulotomy and DBS-ACC. Created with BioRender.com.

The exact mechanism underlying the effect of cingulotomy remains unclear. However, different hypotheses have been proposed. The initial theory, suggested by Foltz and White, was that interruption of the cingulum would restore balance to the limbic system.^28^ A similar theory is supported by Friebel *et al.*^29^ as they propose that pain relief is caused by damaging a hyperactivated ACC, which increases the affective dimension in patients with chronic pain. Yen *et al.*^30^ suggest that destruction of the cingulum bundle and/or cingulate cortex interrupts the connection of the afferent pain fibres for the affective component of pain, leading to a change in the perception of pain. It has been argued that patients may be less able to fixate on their pain, becoming more easily distracted and therefore habituated towards that pain.^31^ Patients’ pain might habituate with time, making them gradually less aware of the pain and reducing pain generated behaviour. This could explain the discrepancy between subjective rating of pain reduction and pain related behaviour.^4,32^ When reviewing the fibre tracts in the cingulate bundle, cingulotomy lesions in the ACC portion will likely affect fibres that cross the cingulate bundle as well as other (widespread) connections in this part of the cingulate bundle.^33^ It has been suggested that the effectiveness of cingulotomy in depression depends on disruption of amygdala fibres, which are connected to more subgenual portions of the cingulate bundle.^34^ Lindsay *et al.*^35^ describe the involvement of a similar network and argues that cingulotomy reduces chronic pain through descending pain modulation of the periaqueductal grey (PAG), amygdala and dorsolateral prefrontal cortex (dIPFC). Another study suggests that the beneficial effects of cingulotomy might be due to its effect on the salience network (Fig. 2).^36^

#### Surgical technique and targeting

**Figure 3:**
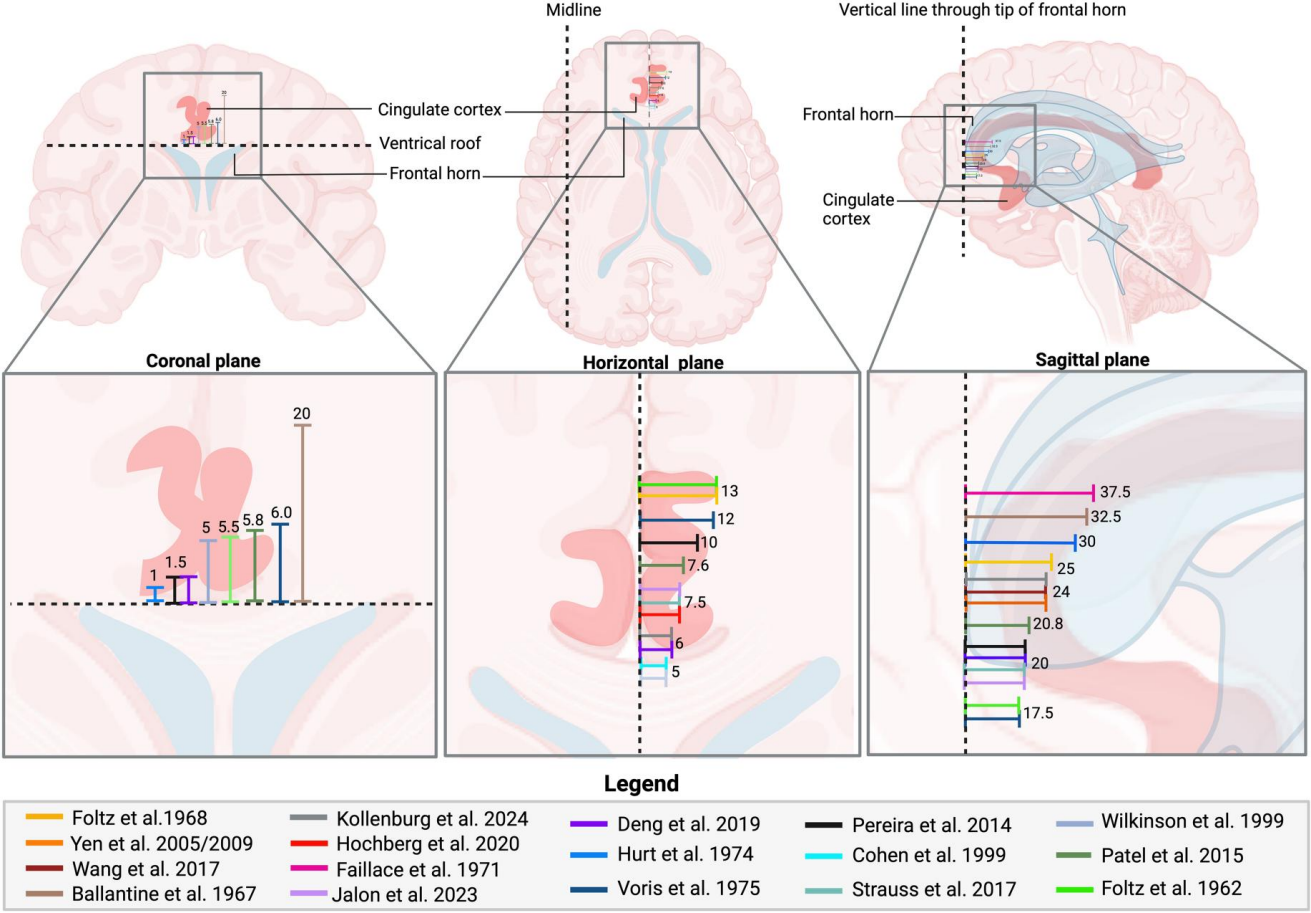
**Targeting in cingulotomy overview of targets reported for cingulotomy, depicted in the coronal, horizontal and sagittal plane.** Please note that the distances along the dashed line, between the solid lines representing the different studies do not indicate actual differences but were displayed in such manner for the purpose of a clear graphical overview. Created with BioRender.com.

The frontal horns are mostly used as a reference for targeting the exact location for lesioning (Fig. 3). Though many clinicians use the frontal horns for guidance, the location of the target varies as different distances from the reference have been reported. The distances from target to the coronal plane above the ventricle roof, sagittal plane posterior to the tip of the frontal horn and horizontal plane from the midline are reported to be 1–20, 5–13 and 17.5–37.5 mm, respectively (Table 1, Supplementary Table S1, Fig. 3). Over time, many techniques have been used as guidance to localize the targets for cingulotomy, starting with ventriculography^14,16,25,28,32^ and fractional pneumocephalography^14^ in the early days of functional neurosurgery, eventually progressing to the use of CT^32^ and MRI^3,4,24,30,36-44^ (Supplementary Table S1). Though all of these techniques have been used in the past, recent studies often mention a preference for MRI as it is thought to be more precise and safe, allowing for direct visualisation of the cingulate gyrus for stereotactic localisation of lesions pre- and post-surgery.^38^ Once visualisation has allowed for accurate localisation of the target, burr holes are created and lesioning is performed using a thermocoagulation electrode. Authors describe the use of different radiofrequency electrodes with tips ranging from 4 to 10 mm (Supplementary Table S1). In addition, clinicians use different parameters for lesioning with temperatures reaching from 45 degrees Celsius (°C) up to 80°C for 15–120 s per lesion. It appears that since 2000, all studies used radiofrequency thermocoagulation lesioning at 80°C for 60–90 s.^24,30,36,40-43,45,46^ In contrast, articles published before 2000 report lesioning at lower temperatures (45–75°C) for 15–60 s (Table 1, Supplementary Table S1).^28,38,39^ The final lesion size is not regularly described but the mean lesion size is reported to be 0.855–1.44 cm.^34,41^ Furthermore, both unilateral and bilateral lesioning have been mentioned with the amount of lesions varying between one and five per side (Table 1, Supplementary Table S1).

**Table 1:** Overview of studies on cingulotomy for neoplastic and non-neoplastic (chronic) pain; target location and technique

Study	No. of lesions	Coronal plane, mm above ventricle roof	Sagittal plane; mm posterior to tip of frontal horn	Horizontal plan; mm from midline	Temperature	Lesioning time	Lesion size
Foltz and White^14^	Unilateral (5 patients), bilateral (11 patients). In first 6 patients 3 lesions were made anteriorly and posteriorly from the initial point.	5.5 (5–6)	17.5 (10–25)	13	N/A	20–30 s	N/A
Ballantine *et al.*^16^	Bilateral, 2 per side	20 and second lesion 10 below first lesion	32.5 (25–40)	7.5 (5–10) lateral to midline	2–8 W	15–30 s (later 60 s)	Lesion extent about 2cm
Foltz and White^28^	Bilateral	95–100 posterior to nasion	25, in case of second lesion further posteriorly	13	45–50°C	N/A	Approximately 1.2 cm in diameter
Faillace *et al*.^47^	Bilateral	N/A	37.5 (35–40)	N/A	N/A	N/A	N/A
Hurt and Ballantine^25^	Bilateral, 2 per side	20 in height 1 mm above roof	30 (20–40)	N/A	8W	60–75s	Transverse diameter about 2 cm with 60s
Voris and Whisler^48^	Bilateral, 2 per side	6	17.5 (10–25) (usually 13 and 17)	12	N/A	N/A	N/A
Hassenbusch *et al*.^38^	Bilateral, 1 per side	N/A	N/A	N/A	75°C (1 patient 85C)	60 s (1 patient 90 s)	5 × 15 mm cilinders
Pillay and Hassenbusch^39^	Bilateral, 1 per side	N/A	N/A	N/A	75°C	60s	5 × 15 mm cilinders
Wong *et al*.^37^	Bilateral, 1 per side	N/A	N/A	N/A	N/A	N/A	N/A
Cohen *et al*.^4^	Bilateral, 1 per side	N/A	N/A	Slightly lateral to midline, 5 mm diameter	N/A	N/A	Mean total volume lesions: 855.4 ± 61.2 mm^3^ (mean ± SD)
Wilkinson *et al*.^32^	Bilateral, 2 per side	5, second lesion 10 mm withdrawal	N/A	5	80°C	120s	N/A
Yen *et al*.^30,42^	Bilateral, 1 or 2 per side	Second lesion 3 mm above first	24	N/A	80°C	80s	N/A
Tsai *et al*.^44^	Bilateral	N/A	N/A	N/A	N/A	N/A	N/A
Pereira *et al*.^45^	Bilateral, 2 per side	1.5 (1–2), second lesion withdrawing 10 mm	20	10	80°C	90s	Effective lesion: cylindrical, 20 mm high, 8–10 mm diameter
Patel *et al*.^41^	Bilateral, 2 per side	Mean 5.8, second 10–20 mm above first lesion	Mean 20.8	Mean 7.6	10.0W (10–11) (median, range)	Mean 240 s per cingulum	Mean lesion size 1440 mm^3^
Strauss *et al*.^40^	Bilateral, 1 lesion (10 mm tip) or 2–3 adjacent lesions in each trajectory (2 mm tip)	N/A	20 (24 and 16)	7.5 (7–8)	80°C	80s	N/A
Wang *et al*.^43^	Bilateral, 1 per side (3 for reoperation)	N/A	24 (reoperation: 5 mm anterior and posterior to initial lesions)	N/A	30W, 80°C	60 s (reoperation 90 s)	Right side median 7.35 mm (4.3–8.5), left side median 7.05 mm (4.6–9.2)
Deng *et al*.^24^	Bilateral, 5 per side	1.5 (1–2), each lesion 4 mm above the other to result in ‘one’ 20 mm long lesion	20, third lesion 5 mm posterior to first	6 (5–7), second lesion 5 mm lateral to first, third lesion 2 mm lateral to first	80°C	60s	5 mm diameter, 20 mm in length
Hochberg *et al.*^46^	Bilateral, 2 per side	N/A	20 (24 and 16)	7.5 (7–8)	80°C	60s	N/A
Jalon *et al*.^36^	Bilateral, 1 lesion (10 mm tip) or 2–3 adjacent lesions in each trajectory (2 mm tip)	N/A	20 (24 and 16)	20 (24 and 16)	80°C	80s	N/A
Kollenburg *et al*.^3^	Bilateral, 2 lesions on the left and 3 on the right side	N/A	24	6 lateral to midline	75°C	60s	N/A

N/A, not available.

#### Pain relief

Our data analysis shows that following cingulotomy, significant pain relief is obtained in 59–77% of patients with various non-neoplastic and neoplastic pain syndromes. The ORR for patients with neoplastic and non-neoplastic pain is 43–64% and 51–53%, respectively at ≤6 months follow-up. With regard to the long-term efficacy, the ORR for both groups is 82% (9/11) and 76% (90/118), respectively at ≥12 months follow-up (Table 2). Various studies found a decrease in efficacy at longer follow-up moments,^25,30,32,46,48^ whereas in other articles, the efficacy was maintained in the long-term (Table 3, Supplementary Table S2).^3,24,30,49^ Interestingly, patients undergoing cingulotomy described the pain to not ‘worry them’ and to be ‘not particularly bothersome’ after surgery.^14,50^ In the studies reviewed here, 23 reoperations were performed. Despite the lack of detail in some reports, a positive effect after reoperation was found in 53% (9/17) of patients (calculated from Supplementary Table S2).^14,25,28,32,40,41,43^

**Table 2:** Overall response rate per pain aetiology

Follow-up moment	All patients	Neoplastic pain	Non-neoplastic pain
Cingulotomy^a^			
≤3 months	59% (90/152)	64% (63/99)	51% (27/53)
>3− ≤ 6 months	49% (43/88)	43% (15/35)	53% (28/53)
≥12 months	77% (99/129)	82% (9/11)	76% (90/118)
DBS-ACC^a^			
≤6 months	59% (10/17)	N/A	59% (10/17)
≥12 months	57% (8/14)	N/A	57% (8/14)

Outcomes presented in this Table have been calculated using data from Supplementary Tables S2 and S3.

^a^Please note that outcomes obtained from reports lacking sufficient details on the definition of responders and/or indications of their subjects, as well as studies reporting a follow-up, which could not be categorized in the table above (e.g. studies reporting a single response rate for a follow-up range covering various time categories), were not included in the calculations above. In case various follow-up moments were described within the same study, only the amount of responders reported at the last follow-up within each time category was included for the calculations.

**Table 3:** Overview on studies of cingulotomy for neoplastic and non-neoplastic (chronic) pain; patients, effect and complications

Study	No of patients	Pain	Follow-up time	Definition responders	Results
Foltz and White^14^	16	Neoplastic (6); Non-neoplastic (10)	4 d–7 y	Good and/or excellent results	Neoplastic: 1 m 4/6, 5 m 1/2 good–excellent pain relief; non-neoplastic: 3 m 6/10, 5 m 6/9, 2 y 4/6, 7 y 1/1 good-excellent pain relief
Ballantine *et al*.^16^	69	Neoplastic (12); Non-neoplastic (57)	3 m–4 y	N/A	N/A
Foltz and White^28^	35	Neoplastic (11); Non-neoplastic (24)	1–9 y	Good and/or excellent results	Neoplastic: 9/11 good-excellent pain relief; Non-neoplastic: 18/24 good-excellent pain relief
Faillace *et al*.^47^	9	Neoplastic (7); Non-neoplastic (2)	3 d− > 2 y	N/A	Neoplastic: 3/7 pain relief; Non-neoplastic: 1/2 pain relief
Hurt and Ballantine^25^	68	Neoplastic (32); Non-neoplastic (36)	4 d–9 y	≥40% reduction in pain intensity	Neoplastic: ≤ 3 m 18/32 (40–100% improvement), >3 m 2/9 (70–90% improvement); Non-neoplastic: ≤ 3 m 16/36 (40–100% improvement), >3 m 16/36 (40–100% improvement); 14/28 reported partial or complete resumption of activity
Voris and Whisler^48^	16	Neoplastic (5); Non-neoplastic (11)	1 m–12 y	N/A	Neoplastic: 1–12 m 5/5 pain relief; Non-neoplastic: 1–12 m 8/11, 1–3 y 2/11, >3 y 1/11 pain relief
Brown *et al*.^49^	43^a^	Non-neoplastic	1–20 y	Good and/or excellent results^b^	39/43
Hassenbusch *et al*.^38^	4	Neoplastic	2 w–4 m	≥50% reduction in pain medication intake	Immediate: 4/4 pain relief. 3/4 complete pain relief until time of death (2–6 w), 1/4 excellent pain relief at last follow-up (4 m)
Pillay and Hassenbusch^39^	10	Neoplastic (8); Non-neoplastic (2)	6 m–1 y	Good and/or excellent results	Neoplastic: 6 m 4/8 excellent; Non-neoplastic: 1 y 1/2 good pain relief.
Wong *et al*.^37^	3	Neoplastic	N/A	≥50% reduction in pain medication intake	2/3
Cohen *et al*.^4^	12	Non-neoplastic	1 y	N/A	1 y 8/12 modest pain relief
Wilkinson *et al*.^32^	23	Non-neoplastic	1–15 y	≥30% reduction in pain score	2–9.5 y: 20/23
Yen *et al*.^42^	22	Neoplastic (15); Non-neoplastic (7)	1 w–1 y	≥30% reduction in pain intensity^b^	Neoplastic: 1 w 12/15, 1 m 10/15, 3 m 7/12, 6 m 5/10; Non-neoplastic: 1 w 7/7, 1 m 5/7, 3 m 5/7, 6 m 5/7, 1 y 5/7
Yen *et al*.^30^	10	Neoplastic	3 m	≥30% reduction in pain intensity^b^	1 w 6/10, 1 m 5/10, 3 m 6/10
Tsai *et al*.^44^	2	Non-neoplastic	N/A	N/A	2/2 reported pain relief
Pereira *et al*.^45^	1	Neoplastic	1–4 m	≥30% reduction in pain intensity	1 m: 1/1; 4 m: 1/1
Patel *et al*.^41^	3	Neoplastic	2 w–4 m	≥50% reduction in pain intensity	2 w 3/3, 6 w 1/2, 4 m 1/1
Strauss *et al*.^40^	13	Neoplastic	1–3 m	≥50% reduction in pain intensity	Immediate 13/13^c^, 1 m 8/11, 3 m 5/7
Wang *et al*.^43^	24	Non-neoplastic	51.5 m (6–77) (median, range)	N/A	Initial median VAS 8, 1 m 3, 3–6 m 4, last follow-up 5
Deng *et al*.^24^	2	Non-neoplastic	18–60 m	≥50% reduction in pain intensity	18 m 2/2, 60 m 1/1
Hochberg *et al*.^46^	23	Neoplastic	3 m	≥30% reduction in pain intensity	Immediate 20/22, 1 m 16/19, 3 m 7/12^c^
Jalon *et al*.^36^	12	Neoplastic	1 m	≥50% reduction in pain intensity	Immediate 9/12, 1 m 9/12
Adams *et al*.^51^	1	Neoplastic	1–3 d	≥50% reduction in pain intensity	1 d 1/1, 3 d 1/1
Kollenburg *et al*.^3^	1	Non-neoplastic	3 y	≥50% reduction in pain intensity	18 w 1/1, 26 w 1/1, 3 y 1/1

CS, case series; CC, case control; CR, case report; CHR, chart review; N/A, not available; d, days; w, weeks; m, months; y, years; h, hours; VAS, visual analogue scale.

^a^Although cingulotomy was performed in all subjects of the study, nine patients received additional lesions in the amygdaloid nucleus and two patients required innominate targets.

^b^For this study only categories of <25%, 25–75% and/or >75% improvements in pain intensity were provided; hence, their 30% response rate might also include some patients with 25–30% improvements.

^c^For these/this value(s), the percentage pain relief was not mentioned for the individual patients.

#### Complications and side-effects

Seizures (3.1%, 13/420) and intracranial haemorrhage (0.7%, 3/420) are the most frequently reported serious adverse events (AEs). Transient and mild AEs most predominantly consist of transient urinary incontinence (>2.1%, >9/420), and akinesia and/or bradykinesia and/or psychomotor slowing (1.9%, 8/420) (Table 4). Some patients also suffered psychiatric complications such as change in affect (>6.9%, >29/420), disorientation (1.9%, 8/420) and confusion (>4.8%, >20/420). In addition, side effects including transient akinesia, bradykinesia, psychomotor slowing, confusion, disorientation and mutism were present, but disappeared after <1 week (Table 4). Though more somatic and psychiatric complications have been reported, these were much lower in prevalence (<1.7%) (Table 4). Lastly, mortality due to the procedure has not been described in the total of 420 patients included in the current analysis, except for two cases of suicide postoperatively (0.5%) described by Foltz and White (Supplementary Table S2).^52^ However, these patients were already at risk for suicide before the procedure due to the presence of depression, hence making it unclear whether the suicides were related to the cingulotomy.

**Table 4:** Complications and side-effects after cingulotomy over a total of 420 patients

	Complications and side effects	Number of patients^a^
Serious	Seizures	13 (3.1%)
	Postoperatively	5 (1.2%)
	Delayed, controlled with medication	4 (1.0%)
	Intraoperatively	2 (0.5%)
	Delayed, not controlled with medication	1 (0.2%)
	Tonic clonic 2 days	1 (0.2%)
	Intracranial haemorrhage	3 (0.7%)
	Hemiparesis	2 (0.5%)
	Prolonged stupor	2 (0.5%)
	Suicide postoperatively (preoperative tendency)	2 (0.5%)
	Complete hemiplegia (preoperative hemiparesis)	1 (0.2%)
	Guillain Barre syndrome	1 (0.2%)
	Ventriculomegaly	1 (0.2%)
Transient and mild somatic	Transient urinary incontinence	>9 (>2.1%)
	Transient akinesia, bradykinesia, psychomotor slowing (<1 week)	8 (1.9%)
	Transient headache (1 week)	>2 (>0.5%)
	Fatigue	2 (0.5%)
	Upper gastrointestinal bleed controlled with medication	2 (0.5%)
	Hypotension (2 days)	1 (0.2%)
	Mild hemiparesis, full recovery	1 (0.2%)
	Reappearance Horner’s syndrome (4 months)	1 (0.2%)
	Transient exacerbation of pain	1 (0.2%)
	Transient hypotension (2 days)	1 (0.2%)
	Hypaesthesia chest wall	Unknown
	Mild elevation in temperature	Unknown
	Transient bowel incontinence	Unknown
Psychiatric	(Transient) change in affect	>29 (>6.9%)
	Transient confusion (<1 week)	>20 (>4.8%)
	Transient disorientation in time (3–5 days)	8 (1.9%)
	Changes in emotional behaviour, personality or cognitive ability	6 (1.4%)
	Attentional impairment	5 (1.2%)
	(Transient) decline in language, executive function or abstract thinking	7 (1.7%)
	Decrement in tapping test	4 (1.0%)
	Transient aphasia (2–5 days)	3 (0.7%)
	Transient mutism (<1 week)	3 (0.7%)
	Decrement in Porteus maze test after 2 months	2 (0.5%)
	Lethargy	2 (0.5%)
	Decline in concentration and recent memory	1 (0.2%)
	Decline in vasoconstrictive skills	1 (0.2%)
	Transient repetitive hand washing (<1 week)	1 (0.2%)

^a^Please note that studies lacking details on the amount of patients having a specific complication and/or side effect, could not be included into the calculations of the overall adverse events rate. Hence, the ‘>’ has been added to some values, indicating that the rate is likely higher but could not be further determined.

### Deep brain stimulation of the anterior cingulate cortex

Although DBS has been proposed over 40 years to treat refractory neuropathic pain, the ACC has only recently been emerging as a neurosurgical target due to the growing appreciation of emotional components of pain.^8^ More commonly used targets for chronic pain include the ventral posterior medial/ventral posterior lateral nuclei, parafascicular-centre median nuclei and periaqueductal/periventricular grey.^53^ DBS-ACC was expected to have similar effects on pain reduction as both techniques use the ACC as neurosurgical target.

#### Mechanism

The initial idea of stimulating the cingulum was to create an effect similar to cingulotomy by functional inactivation of cell bodies.^54^ This is supported by patients reporting a similar effect on the intensity and affective component of their pain.^15^ However, stimulation is different from ablation as stimulation of axons of passage might also have a distant or antidromic effect.^55,56^ Despite the exact mechanism of DBS-ACC being unknown, various studies state that the effects of DBS-ACC can be attributed to its interference with complex pain pathways involving the ACC, PAG and thalamus.^57-60^ Mohseni *et al.*^57^ show the presence of long-term changes in activity of the ACC and PAG following DBS-ACC (Fig. 2). These observations were supported when stimulation of the thalamus and PAG enhanced the activity in the rostral and dorsal ACC.^57-60^ Similarly, research in rodents shows that stimulation of the ACC reduces aversive pain responses by decreased thalamic activity, caused by the stimulation of ACC inhibitory neurons.^8^ Pagano *et al.*^8^ suggest that DBS-ACC reduces GABAergic PAG firing and decreases glutamatergic transmission from thalamic cells projecting to the primary sensory cortex (Fig. 2). Besides, it is also speculated that DBS-ACC might stimulate all structures of the cingulum simultaneously which may augment analgesia.^61^ Further, data also shows increased connectivity between the MCC and posterior insula in patients with chronic pain.^62^ This evidence may support the hypothesis that effects of DBS-ACC can be in part attributed to alterations in inputs arriving the posterior insula. Understanding of the viscero-motor cortical areas, like the ACC and insula, may therefore add a more dynamic exploratory framework on how DBS-ACC could change pain processing, without directly affecting its sensory discriminative component.^63^ The strength in connectivity of the fibres from the activated tissue around the electrodes also seems important, as tractography shows that these predict the effects on pain relief.^56^ Strong connectivity to the precuneus area seems to predict unsuccessful outcomes, whereas connectivity to the thalamus, insula and brainstem through the medial forebrain bundle is related to successful outcomes.^56^ These results indicate that the medial forebrain bundle, generally considered to be involved in the integration of reward and pleasure and possibly involved in interindividual differences in pain sensitivity, may also play a role in emotional interpretation of pain.^56,64^ Indirect modulation of this bundle by DBS-ACC may also reduce activity of the PAG and resort in pain relief.^65^

#### Surgical technique and targeting

In all studies, the same electrodes are used and stimulated at 130 Hz. Several voltages ranging from 2.5 to 6.5 V are used for stimulation. For the electrode placement, authors only report usage of preoperative MRI guidance (Table 5, Supplementary Table S3). Besides, all studies use a similar target (20 mm posterior to anterior tip of frontal horns of lateral ventricles) and placed the DBS electrode in the cingulate bundle with the deepest contact located in the corpus callosum (Table 5, Supplementary Table S3).^15,61,66,67^ All authors performed bilateral implantation (Table 5, Supplementary Table S3).

**Table 5:** Overview of studies on DBS-ACC for chronic pain; patients, effect and complications

Study	No. of patients	Diagnosis	Follow-up time	Target	Stimulation parameters	Definition responders	Results	Complications/side effects
Spooner *et al*.^54^	1	Spinal cord injury C4	2–4 m	Bilateral, 20 mm posterior to anterior margin of the lateral ventricles in the midsection of the gyrus	130 Hz	≥30% reduction in pain intensity and/or pain medication intake	2 m: 1/1 (VAS 50% decrease and pain medication use 11% decrease) 4 m: 1/1 (VAS 63% decrease and pain medication use 56% decrease)^a^	None
Boccard *et al*.^61^	1	Traumatic brachial plexus injury	2 y	Bilateral, 20 mm posterior to anterior tip of frontal horns of lateral ventricles, contacts in cingulum bundle, deepest contact in corpus callosum	2.5 V, 130 Hz, 330us; later on 4 V, 130 Hz, 450us	≥30% reduction in pain intensity	1 y: 1/1 (VAS 40% decrease) 2 y: 1/1 (VAS 55% decrease, MPQ 42% decrease, SF-36 2.1% increase, EQ-5D unchanged)	2 y: Stroop task improvement, increase in apathy and executive dysfunction
Boccard *et al*.^67^	16	6 FBSS, 4 PSP, 3 brachial plexus injury, 1 cervical SCI, 1 head injury, 1 unknown chest pain	13.2 m (1–36 m) (mean, range)	Bilateral, 20 mm posterior to anterior tip of frontal horns of lateral ventricles, contacts mostly in cingulum bundle, deepest contact in corpus callosum	Mean and range: 5 V (4–6.9), 128.6 Hz (120–130), 450us across all four contacts	≥30% reduction in pain intensity	5/11 [VAS 24.5% decrease, SF-36 7.3% increased (however physical functioning increased significantly), EQ-5D 20.3% improvement, MPQ 16.0% improvement]	Infection (1, removed), increased pain postoperatively (3/11)
Boccard *et al*.^15^	24	9 PSP, 6 FBSS, 3 brachial plexus injury, 2 SCI, 2 road traffic accident, 1 head injury, 1 unknown chest pain	39.2 m (24–65 m) (mean, range)	Bilateral (21/24 patients), only left (2), only right (1), 20 mm posterior to anterior tip of frontal horns of lateral ventricles, contacts mostly in cingulum bundle, deepest contact in corpus callosum	Range: 4–6.5 V, 130 Hz, 450us	≥30% reduction in pain intensity	6 m 7/11, 12 m 5/8, 24 m 0/4, 36 m 2/8 Two patients not implanted after trial period (no pain relief) 6 m: NRS 45% decrease, MPQ 36% decrease, EQ-5D 21% decrease, SF-36 physical functioning 54.2% improvement 10/12 (83.3%) implanted patients reported substantial pain relief Generalized loss of efficacy at longer follow-up Mild improvement in reasoning (1/8), verbal memory (2/8), Stroop (2/8), FAS (1/8), semantic fluency (2/8) and significant improvement in visual memory (1/8), Stroop (1/8), psychomotor speed (1/8) and mood (1/8)^b^	Infection (5, removed), broken leads (2, 1 removal), stimulation induced seizures (2), de novo stimulation-induced epilepsy (2, even continuing after cessation stimulation), 2 other seizures (1 alcohol withdrawal and in one events ceased after explantation due to infection) Mild decline in semantic fluency (3/8 tested), psychomotor speed (2/8), FAS (1/8) and significant decline in Stroop (1/8)
Levi *et al*.^66^	5	Thalamic ischaemic lesions causing hemi-body and hemi-facial pain	18 m	Bilateral, 20 mm posterior to the anterior tip of the frontal horns of the lateral ventricles, contacts mostly in cingulum bundle, deepest contact in corpus callosum	Mean and range: 4.5 V (4–5.5), 130 Hz, 450us on all four leads	≥30% reduction in pain intensity	6 m: 2/5 (NRS 38% decrease, MPQ 16.2% decrease, SF-36 24% increase, EQ-5D pain 23% decrease) 18 m: 2/5 (NRS 35% decrease, MPQ 17.1% decrease, SF-36 4.1% increase, EQ-5D pain 23% decrease)	None

CR, case report; CS, case series; N/A, not available; d, days; w, weeks; m, months; y, years; h, hours; NRS, numeric rating scale; VAS, visual analogue scale.

^a^As the preoperative VAS score was not mentioned, percentages were calculated based on the average VAS scores measured at 2 and 4 m postoperatively when the stimulator was turned off.

^b^Due to the lack of individual patient data, these response rates were calculated based on the box-plots provided in the article.

#### Pain relief

Despite none of the studies reporting data for patients with neoplastic pain, the ORR of DBS-ACC for non-neoplastic pain is 59% (10/17) at ≤6 months. With regard to the long-term outcomes, the ORR is 57% (8/14) at ≥12 months follow-up (Table 2). Both case reports included in this review, observe significant pain reduction following implantation.^54,61^ For a follow-up period of 6 months, the NRS score decreases by 38–45%, and after 12 and 24 months, the VAS score decreases by 40% and 42%, respectively^15,61,66^ (calculated from data in Supplementary Table S3). Despite some studies reporting that the efficacy of DBS-ACC is improves or remains stable over time,^66^ Boccard *et al.*^15^ observed a generalized loss of efficacy at longer follow-up. Though most of the patients receiving DBS-ACC reported sufficient pain relief, 2/47 (4.3%) patients did not undergo implantation after a trial period. Furthermore, 6/47 (12.8%) patients had the DBS system removed at a later stage due to insufficient pain relief and/or unwanted AEs (calculated from Supplementary Table S3). Besides, it is remarkable that some of the patients receiving modest pain reduction, asked for the stimulator to be turned ON as well as implanted pulse generator replacement.^66^ Patients receiving DBS-ACC use the same expressions as the patients of Foltz and White in 1962 after cingulotomy, describing their pain as ‘less bothersome’.^15,53^

#### Complications and side-effects

Whereas the study by Levi *et al.*^66^ do not report any side-effects following DBS-ACC, other reports observed several complications including infection (12.8%, 6/47) and lead breakage (4.3%, 2/47), leading to removal of the DBS implants. DBS-ACC, also induced seizures in 8.5% (4/47) of patients, with two subjects having de-novo epilepsy after long-term stimulation, not controlled by antiepileptic medication (Table 5, Supplementary Table S3). The case study by Boccard *et al.*^61^ observes an increase in apathy and executive dysfunction. Lower incidences of decline in semantic fluency (6.4%, 3/47), psychomotor speed (4.3%, 2/47), FAS (2.1%, 1/47), executive dysfunction (2.1%, 1/47) and Stroop (2.1%, 1/47) have also been reported (Table 5, Supplementary Table S3). Despite the presence of these AEs, the authors of the case study did not find an association between the effect of stimulation and neuropsychological outcomes (e.g. mood, psychomotor speed, visual or verbal memory).^15^

## Discussion

Chronic pain places a huge burden on patients’ lives, especially for those refractory to conventional pharmacological or (minor) invasive pain management strategies.^5^ Hence, there is a need for alternative approaches. Several neurosurgical strategies such as cingulotomy and DBS-ACC have been used to treat chronic pain. Especially for chronic intractable pain, the cingulum appears to be an important target, as, unlike most treatments, DBS-ACC and cingulotomy affect other factors involved in suffering, including emotional reaction, attention, processing and perception of pain, rather than pain intensity alone.

### Cingulotomy

The current results support that cingulotomy is an effective last-resort therapy for chronic pain, causing substantial pain relief in 51–82% of patients (Table 2). Similarly, Sharim and Pouratian^10^ report a response rate of >60%. Our findings show an immediate response to cingulotomy at ≤ 1 month follow-up (Table 3).^14,30,36,38,40-42,46,51^ In contrast, studies on obsessive compulsive disorder (OCD) reported a delayed effect of >6 m following cingulotomy.^68-70^ It is suggested that neuronal reorganisation of white matter structures might be responsible for the delayed clinical improvements seen in patients with OCD at longer follow-up.^70^ Interestingly, following cingulotomy, patients with neoplastic pain were found to have a higher ORR after ≤ 3 months follow-up as compared with the group suffering non-neoplastic pain (Table 2). This might be explained by findings showing that cancer related somatic and/or hereditary factors contribute to neuronal degeneration at the lesion site, thus enhancing the effects of cingulotomy.^71^ However, caution should be taken as the difference between both groups is rather small. Furthermore, some studies observe a decrease in efficacy at longer follow-up.^25,30,32,46,48^ The decrease in efficacy may be correlated to regeneration of neurons in the lesion site and surrounding areas and recovery of activity in brain networks involved in the perception of pain. The effect of cingulotomy has shown to be mediated through a decrease in the functional connectivity within the salience network, which may recover.^3,36^ Insufficient lesioning may enhance the chance of neuronal regeneration and, thus, possibly lowers the power of long-term effect, eventually leading to a reoperation.^10^ To prevent insufficient lesioning, Strauss *et al.*^40^ suggest that adding a second lesion bilaterally would enhance long-term effects as neuronal regeneration is minimalized. Although the number of lesions varies among studies, there seems to be a trend to make (at least) two lesions and a consensus on doing this bilaterally, regardless whether pain is unilateral or bilateral. Long-term functional MRI studies in patients with a recurrence of pain after cingulotomy, will aid in evaluating the hypothesis on neuronal regeneration.

The current review also confirms the absence of a standardized protocol for cingulotomy as surgical aspects such as lesion size, target and imaging vary among studies. With regard to lesion size, it might seem reasonable to think that a bigger lesion is more effective due to enhanced neuronal destruction in tissues interacting with pain. Evidence for this hypothesis is that studies show that reoperations, in which the total lesion volume is enlarged, causes significant pain relief in 53% of the failed cases (Supplementary Table S2). However, Steele *et al.*^72^ argue against this in the case of depression, suggesting that rostral extension, rather than enlargement of the ACC lesion, is responsible for the therapeutic effect seen in reoperations. Though Steele and colleagues report an optimal lesion size of 1000–2000 mm^3^ for depression, it remains a matter of ongoing debate whether there might be such an optimal lesion size when performing cingulotomy for chronic pain as only few studies reported the lesion size overall. A correlation between lesion size and effect is thus hard to prove due to the lack of sufficient data. It is also noteworthy that the target for lesioning greatly varies among studies as the distance from the reference points differed up to 20 mm (Fig. 3). Though this variability was partially expected due to the presence of anatomical variations, the lack of standardisation is also thought to be responsible for this phenomenon. Even though the optimal target location remains elusive, several studies argued that more anterior lesions were correlated with better outcomes in treating chronic pain as well as depression, owing to the extensive involvement of the ACC in emotional processing as well as higher density of afferent- and efferent fibres in this area, as compared to more posterior regions (e.g. parahippocampal area).^10,12,13,33,72^

Following cingulotomy, various AEs have been reported, most of which disappeared within 1 week. Its destructive nature, limited understanding of the cingulate cortex and absence of a standardized surgical approach likely contributed to the occurrences of AEs. A decline in language following cingulotomy was reported by a subset of patients. Upon reviewing of the previous reports, aphasia and/or inappropriate language appears to be present in 2% (4/236) of patients with chronic intractable pain undergoing cingulotomy.^10^ Despite the absence of an explanation for this AE, the presence of aphasia pre-surgery, possibly contributes to this occurrence.^3^ Moreover, a ‘change in affect’, was also experienced by some patients postoperatively. However, as ‘change in affect’ is often not defined and could include both negative (e.g. experiencing negative emotions) and positive (e.g. improved pain behaviour) changes, it makes it questionable to what extend this AE truly caused unwanted reactions. Considering the extensive role of the cingulum in emotional processing and/or pain behaviour, alterations in affect related to nociception were also expected. The effect of cingulotomy on pain processing is even described to be largely responsible for the beneficial effects seen in patients with chronic intractable pain.^13^ Interestingly, no serious AEs were reported since 2000. This might be caused by recent improvements in imaging techniques making targeting more precise, and cingulotomy a safer and more effective procedure.

### Deep brain stimulation of the anterior cingulate cortex

DBS-ACC has shown to be an effective treatment for chronic pain when less-invasive options have failed, as postoperative results show an ORR of 57–59% (Table 2). The study outcomes on DBS-ACC are consistent with one another as outcomes on pain intensity were similar. This is likely caused by similar surgical approaches to DBS-ACC and 3/5 studies coming from the same group.^56,61,67^ Though these factors constrained the determination of the optimal surgical technique, it emphasizes the importance of a standardized surgical protocol in the achievement of steady clinical outcomes. In contrast to cingulotomy, no reports are published on DBS-ACC for neoplastic pain. This analysis also shows a general loss of efficacy at longer follow-up in some patients undergoing DBS-ACC.^15^ A plausible hypothesis for this decrease could be DBS-induced plasticity after long-term stimulation. A similar phenomenon is also seen in patients receiving DBS for an essential tremor^73,74^ and might therefore also provide an explanation for the loss of efficacy over time in patients with chronic pain. Besides, patients usually forget about their initial baseline pain and quickly adapt to the new situation, consequently leading to patients reporting higher pain scores after longer periods of stimulation. Further, glial scarring around the electrode may also be involved in the loss of efficacy on the long-term.^15,75^ However, as most reports do not include a long-term follow-up, it remains elusive whether the effects of DBS-ACC improves or diminishes on the long-term. Moreover, only five articles were available for DBS-ACC, hence more research should be performed to properly evaluate the (long-term) clinical efficacy of DBS-ACC for refractory non-neoplastic and neoplastic pain.

Following DBS-ACC, the occurrence of seizures in a subset of patients is reported. Unlike for DBS-ACC, induction of de novo seizures has not been reported for other targets of DBS.^10^ This might imply an epileptogenic role of the cingulum.^66^ Further, seizures have also been reported for motor cortex stimulation and stimulation of other cortical areas.^76,77^ Despite the occurrence of various other AEs, DBS-ACC is considered to be a safe procedure as complications were most predominantly technical (e.g. broken leads) with relative low incidences of biological AEs.

### Cingulotomy versus DBS-ACC

#### Surgical technique and mechanism

Current results show the presence of similarities in the surgical technique of cingulotomy and DBS-ACC. However, as both approaches require different surgical tools (e.g. tasker RF probe versus electrode), with DBS-ACC being more recently developed, differences between both techniques can also be expected. With regard to lesioning, despite reports on both techniques claiming to use the ACC, targets appear to differ from one another. It seems that for cingulotomy the anterior medial cingulate cortex (aMCC) and for DBS-ACC, the cingulate bundle rather than the cingulate cortex is targeted. The uncertainties on the neurosurgical target can be attributed to the absence of clear borders between the cingulate cortex, bundle and its subdivisions.^13^ Though the cingulate bundle and ACC contain similar fibres, additional fibres pass through the cingulum bundle, hence different outcomes can be expected for these targets.^33^ Future studies focusing on connectivity, using diffusion tensor imaging of the lesion site and DBS location, will aid in defining the optimal surgical target for cingulotomy and DBS-ACC.^13^

Furthermore, the mechanism underlying cingulotomy and DBS-ACC differ from one another as lesioning is fundamentally different from stimulation. Whereas cingulotomy affects the salience network^36^ and circuits connecting the PAG, amygdala and DlPFC,^35^ DBS-ACC has been described to mostly act upon its connections towards the PAG.^8^ Stimulation might mimic the effect of destruction, but might also has an effect via the modulation of more remote brain structures within the pain matrix due to the effect on passing axons.^55^ When considering both mechanisms in more detail, lesioning creates destruction and thus irreversible inhibition of neurons whereas DBS allows for reversible stimulation and/or inhibition of neurons (Fig. 2). It remains unclear why some neurons are activated and others inhibited upon stimulation; however, neurophysiological differences between neurons and neurotransmitters are thought to be responsible for this phenomenon.^78^ It is also noteworthy that the area affected by stimulation is often smaller compared to lesioning. The electrodes used for DBS-ACC are approximately 1.27 mm in diameter whereas lesions created with cingulotomy can reach diameters up to 1.2 cm (Tables 1 and 5). With an increased neuronal coverage, it can be argued that cingulotomy might be more effective, however also carries higher risk to permanent AEs as compared to DBS-ACC. Despite the presence of differences between both techniques, which should be considered carefully when selecting a treatment, no research has been performed on the comparison of both techniques. Hence, future research comparing cingulotomy and DBS-ACC would be valuable.

#### Clinical implementation

Next to considerations related to the surgical technique, additional factors should be examined when deciding upon the best indications for both neurosurgical options. Overall, DBS-ACC is considered to be more beneficial for patients with a longer life expectancy when reviewing the financial costs and long-term effects of this technique. Stimulation is more costly due to the need of more and advanced materials such as an implantable pulse generator, leads and electrodes. Besides, DBS-ACC gives the opportunity for personalized stimulation, which might prohibit acquisition of optimal results on the short-term as the optimal settings are often determined after months of stimulation. Considering these arguments, it makes non-neoplastic patients an ideal candidate for this approach as these often have longer-life expectancies as compared to those suffering neoplastic pain. Due to the lack of clinical evidence for DBS-ACC, cingulotomy can be of great interest for patients with neoplastic pain, especially when considering the low costs of the approach and short-life expectancy of these patients. Added to that, patients with neoplastic pain might accept any permanent complications with greater ease due to their short life-expectancy.

#### Transcranial magnetic stimulation

Aside from cingulotomy and DBS-ACC, the ACC has also been explored with non-invasive neuromodulation techniques such as transcranial magnetic stimulation (ACC-TMS).^79^ In ACC-TMS, a brief electrode current is passed through a magnetic coil, allowing for stimulation of the ACC by generating a brief, high-intensity magnetic field.^80^ Evidence suggests that ACC-TMS may cause significant improvements in patients with mood disorders and experimental pain settings.^81^ Galhardoni *et al*.^65^ assessed the efficacy of ACC-TMS in patients with central neuropathic pain and observed significant improvements in anxiety scores but not in pain interference with daily activities, pain dimensions, neuropathic pain symptoms, mood, medication use, cortical excitability measurements or quality of life. Another article by Tzabazis *et al.*^82^ observed a 43% reduction on NRS score in patients with fibromyalgia, but only when operated at 10 Hz. Variability in outcomes for ACC-TMS and chronic pain may not only be contributed to differences in stimulation settings and variability in pain origin, but also to location of the target. To illustrate, it is suggested that brain targets >20 mm deep, including the ACC, cannot be properly stimulated with conventional coils, except for angulated coils.^83^ Furthermore, it is also suspected that certain forms of ACC-TMS, may not be selective to the ACC as it produces a magnetic field that can potentially induce currents in a wider range of brain regions, hence why it remains uncertain if its effect on pain and mood is induced by stimulation of the ACC itself.^84^ Considering these arguments, invasive stimulation and/or lesioning of the ACC with cingulotomy and/or DBS-ACC may be preferred in certain cases, however should be further investigated.

### Strengths and limitations

Even though measures were taken to optimize outcomes of the current analysis, comparison of the overall efficacy of DBS-ACC with cingulotomy remains challenging. This can be partially attributed to the limited data availability of cingulotomy and DBS-ACC for chronic pain. It should also be noted that positive-result bias might have been present as authors often tend to only report research with positive outcomes and minimal occurrence of AEs. Another limitation that should be considered is the large time span (1957–2024) in which articles were published. The advancements in technology and knowledge over time have likely affected the interpretation and thus the conclusions drawn by authors. With regard to the reported AE rates, one should realize that though these were calculated to the best of our expertise, additional AEs might have been present as not all authors analysed the same cognitive, emotional and/or physical parameters perioperatively. Further, evaluation of the efficacy mostly consisted of the standard pain assessment scores. However, as cingulotomy and DBS-ACC are thought to alter the affective component of pain, standard pain assessment scores might underestimate its effect as pain intensity but not its emotional component is measured.^10^ Another issue concerns the lack of control groups as most studies were observational and retrospective. Consequently, the placebo effect might reduce the effect size of cingulotomy and DBS-ACC. Besides, variability and lack of detail with regard to the follow-up moments and the exclusion of patients in-between the measurements also complicated the current analysis. We aimed to minimize the effects of discrepancies in follow-up moments by dividing the follow-up moments into various groups including ‘ ≤ 3 months’, ‘>3– ≤ 6 months’ and ‘≥12 months’. However, as some studies defined a single response rate for a wide follow-up range, some outcomes could not be categorized into these groups, consequently affecting the ORRs. Furthermore, in case various dates were reported by the same study, only the last follow-up moment was included in each category. This could have led to an underestimation of the overall outcomes of cingulotomy and DBS-ACC as various studies found a loss in response of the longer term (Tables 3 and 5). In addition, fluctuations over time due to the natural course of disease, heterogeneity in study population and authors mentioning terms like ‘noncerebral traumatic injury’, without describing what these injuries consisted of^4,16,30,47,48^ also complicated the current analysis. It is noteworthy that none of the included studies provided a definition for responders. Hence, using a definition for responders is also considered a major strength of the current study. Nevertheless, outcomes should be taken with caution as our responder definition includes various pain assessments (e.g. VAS, NRS, pain medication intake) and because authors often poorly defined categorical classifications such as ‘significant’.^42,49^ The lack of standardisation in response definition and pain assessment likely contributes to the subjectivity of the study outcomes. We were aware of this before the analysis; however, as only limited studies were available on both techniques with a lack of standardisation in pain assessment among authors, this could not be prevented. Another factor complicating data analysis with the ORR is the lack of individual subject data, as some studies only reported average pain scores for the whole group instead. Hence, to optimize the analysis of data, additional calculations were performed, allowing for the conversion of grouped data to estimations of the individual data. As the original individual patient outcomes were not available, the calculated values could slightly deviate from the original data.

Nevertheless, calculating individual patient data and defining responders manually for each study provides uniqueness to this article as the ORRs of cingulotomy and DBS-ACC for chronic intractable pain have not been previously evaluated to such extend and provide valuable insights confirming the potential of the cingulum as a neurosurgical target for chronic intractable pain. Due to the presence of limitations in the current analysis and most studies being observational, future clinical trials with a clear responder definition, proper control groups and at least 2 follow-up evaluations, with one being at minimum of 1 year, are necessary to further evaluate the (long-term) effects and possible involvement of placebo effects and/or additional factors in the clinical outcomes of cingulotomy and DBS-ACC.

## Conclusion

Chronic intractable pain causes a major burden on patients’ lives and is also recognized as a major public health problem, in part due to their profound socioeconomic impact. Nevertheless, a subset of patients remain refractory to conventional strategies, hence why alternative strategies like cingulotomy and DBS-ACC should be considered. The current analysis shows that cingulotomy and DBS-ACC are effective last resort strategies for patients with refractory non-neoplastic and neoplastic pain, especially if unaffected emotional component is present. Consensus in targeting of the cingulum is important for any procedure involving the ACC. Future research comparing DBS-ACC and cingulotomy, using a standardized responder definition, as well as outcome measures covering both the physical and emotional components of pain, will allow for further exploration of the cingulum as a target in intractable pain.

## Supplementary Material

fcae368_Supplementary_Data

## Data Availability

Raw data supporting this study and all analyses carried out are included within the article.

## References

[1] Domenichiello AF , Ramsden CE. The silent epidemic of chronic pain in older adults. Prog Neuropsychopharmacol Biol Psychiatry. 2019;93:284–290. 31004724 10.1016/j.pnpbp.2019.04.006 PMC6538291

[2] Hylands-White N , Duarte RV, Raphael JH. An overview of treatment approaches for chronic pain management. Rheumatol Int. 2017;37(1):29–42. 27107994 10.1007/s00296-016-3481-8

[3] Kollenburg L , Kurt E, Arnts H, Vinke S. Cingulotomy: The last man standing in the battle against medically refractory poststroke pain. Pain Rep. 2024;9(2):e1149. 38529477 10.1097/PR9.0000000000001149 PMC10962879

[4] Cohen RA , Kaplan RF, Moser DJ, Jenkins MA, Wilkinson H. Impairments of attention after cingulotomy. Neurology. 1999;53(4):819–824. 10489048 10.1212/wnl.53.4.819

[5] Qassim H , Zhao Y, Ströbel A, et al Deep brain stimulation for chronic facial pain: An individual participant data (IPD) meta-analysis. Brain Sci. 2023;13(3):492. 36979302 10.3390/brainsci13030492 PMC10046035

[6] Racine M . Chronic pain and suicide risk: A comprehensive review. Prog Neuropsychopharmacol Biol Psychiatry. 2018;87:269–280. 28847525 10.1016/j.pnpbp.2017.08.020

[7] Kelly KM , Chung SS. Surgical treatment for refractory epilepsy: Review of patient evaluation and surgical options. Epilepsy Res Treat. 2011;2011:303624. 22937231 10.1155/2011/303624 PMC3420605

[8] Pagano RL , Dale CS, Campos ACP, Hamani C. Translational aspects of deep brain stimulation for chronic pain. Front Pain Res (Lausanne). 2022;3:1084701. 36713643 10.3389/fpain.2022.1084701 PMC9874335

[9] Flouty O , Yamamoto K, Germann J, et al Idiopathic Parkinson's disease and chronic pain in the era of deep brain stimulation: A systematic review and meta-analysis. J Neurosurg. 2022;137(6):1821–1830. 35535836 10.3171/2022.2.JNS212561

[10] Sharim J , Pouratian N. Anterior cingulotomy for the treatment of chronic intractable pain: A systematic review. Pain Physician. 2016;19(8):537–550. 27906933

[11] Santo JL , Arias LM, Barolat G, Schwartzman RJ, Grossman K. Bilateral cingulumotomy in the treatment of reflex sympathetic dystrophy. Pain. 1990;41(1):55–59. 1693763 10.1016/0304-3959(90)91109-V

[12] Stevens FL , Hurley RA, Taber KH. Anterior cingulate cortex: Unique role in cognition and emotion. J Neuropsychiatry Clin Neurosci. 2011;23(2):121–125. 21677237 10.1176/jnp.23.2.jnp121

[13] Kollenburg HA , Green AL, Strauss I, Vinke S, Kurt E. The cingulum: Anatomy, connectivity and what goes beyond. Brain Commun. 2024. 10.1093/braincomms/fcaf048 PMC11824423 39949403

[14] Foltz EL , White LE Jr. Pain “relief” by frontal cingulumotomy. J Neurosurg. 1962;19:89–100. 13893868 10.3171/jns.1962.19.2.0089

[15] Boccard SGJ , Prangnell SJ, Pycroft L, et al Long-term results of deep brain stimulation of the anterior cingulate Cortex for neuropathic pain. World Neurosurg. 2017;106:625–637. 28710048 10.1016/j.wneu.2017.06.173

[16] Ballantine HT , Cassidy WL, Flanagan NB, Marino R. Stereotaxic anterior cingulotomy for neuropsychiatric illness and intractable pain. J Neurosurg. 1967;26(5):488–495. 5337782 10.3171/jns.1967.26.5.0488

[17] Xiao X , Ding M, Zhang YQ. Role of the anterior cingulate Cortex in translational pain research. Neurosci Bull. 2021;37(3):405–422. 33566301 10.1007/s12264-020-00615-2 PMC7954910

[18] Zhao R , Zhou H, Huang L, et al Neuropathic pain causes pyramidal neuronal hyperactivity in the anterior cingulate Cortex. Front Cell Neurosci. 2018;12:107. 29731710 10.3389/fncel.2018.00107 PMC5919951

[19] Lorenz J , Kohlhoff H, Hansen HC, Kunze K, Bromm B. Abeta-fiber mediated activation of cingulate cortex as correlate of central post-stroke pain. Neuroreport. 1998;9(4):659–663. 9559934 10.1097/00001756-199803090-00018

[20] Nagasaka K , Takashima I, Matsuda K, Higo N. Brain activity changes in a monkey model of central post-stroke pain. Exp Neurol. 2020;323:113096. 31682802 10.1016/j.expneurol.2019.113096

[21] Jones AK , Watabe H, Cunningham VJ, Jones T. Cerebral decreases in opioid receptor binding in patients with central neuropathic pain measured by [11C]diprenorphine binding and PET. Eur J Pain. 2004;8(5):479–485. 15324779 10.1016/j.ejpain.2003.11.017

[22] Roosink M , Buitenweg JR, Renzenbrink GJ, Geurts AC, Ijzerman MJ. Altered cortical somatosensory processing in chronic stroke: A relationship with post-stroke shoulder pain. Neurorehabilitation. 2011;28(4):331–344. 21725166 10.3233/NRE-2011-0661

[23] Mustroph ML , Cosgrove GR, Williams ZM. The evolution of modern ablative surgery for the treatment of obsessive-compulsive and major depression disorders. Front Integr Neurosci. 2022;16:797533. 35464603 10.3389/fnint.2022.797533 PMC9026193

[24] Deng Z , Pan Y, Li D, et al Effect of bilateral anterior cingulotomy on chronic neuropathic pain with severe depression. World Neurosurg. 2019;121:196–200. 30315971 10.1016/j.wneu.2018.10.008

[25] Hurt RW , Ballantine HT Jr. Stereotactic anterior cingulate lesions for persistent pain: A report on 68 cases. Clin Neurosurg. 1974;21:334–351. 4370936 10.1093/neurosurgery/21.cn_suppl_1.334

[26] Arnow BA , Hunkeler EM, Blasey CM, et al Comorbid depression, chronic pain, and disability in primary care. Psychosom Med. 2006;68(2):262–268. 16554392 10.1097/01.psy.0000204851.15499.fc

[27] Bair MJ , Robinson RL, Katon W, Kroenke K. Depression and pain comorbidity: A literature review. Arch Intern Med. 2003;163(20):2433–2445. 14609780 10.1001/archinte.163.20.2433

[28] Foltz EL . Current status and use of rostral cingulumotomy. South Med J. 1968;61(9):899–908. 4877691 10.1097/00007611-196809000-00001

[29] Friebel U , Eickhoff SB, Lotze M. Coordinate-based meta-analysis of experimentally induced and chronic persistent neuropathic pain. Neuroimage. 2011;58(4):1070–1080. 21798355 10.1016/j.neuroimage.2011.07.022 PMC8018239

[30] Yen CP , Kuan CY, Sheehan J, et al Impact of bilateral anterior cingulotomy on neurocognitive function in patients with intractable pain. J Clin Neurosci. 2009;16(2):214–219. 19101146 10.1016/j.jocn.2008.04.008

[31] Agarwal N , Choi PA, Shin SS, Hansberry DR, Mammis A. Anterior cingulotomy for intractable pain. Interdiscip Neurosurg. 2016;6:80–83.

[32] Wilkinson HA , Davidson KM, Davidson RI. Bilateral anterior cingulotomy for chronic noncancer pain. Neurosurgery. 1999;45(5):1129–1134. 10549929 10.1097/00006123-199911000-00023

[33] Bubb EJ , Metzler-Baddeley C, Aggleton JP. The cingulum bundle: Anatomy, function, and dysfunction. Neurosci Biobehav Rev. 2018;92:104–127. 29753752 10.1016/j.neubiorev.2018.05.008 PMC6090091

[34] Heilbronner SR , Haber SN. Frontal cortical and subcortical projections provide a basis for segmenting the cingulum bundle: Implications for neuroimaging and psychiatric disorders. J Neurosci. 2014;34(30):10041–10054. 25057206 10.1523/JNEUROSCI.5459-13.2014 PMC4107396

[35] Lindsay NM , Chen C, Gilam G, Mackey S, Scherrer G. Brain circuits for pain and its treatment. Sci Transl Med. 2021;13(619):eabj7360. 34757810 10.1126/scitranslmed.abj7360 PMC8675872

[36] Jalon I , Berger A, Shofty B, et al Lesions to both somatic and affective pain pathways lead to decreased salience network connectivity. Brain. 2023;146(5):2153–2162. 36314058 10.1093/brain/awac403

[37] Wong ET , Gunes S, Gaughan E, et al Palliation of intractable cancer pain by MRI-guided cingulotomy. Clin J Pain. 1997;13(3):260–263. 9303260 10.1097/00002508-199709000-00013

[38] Hassenbusch SJ , Pillay PK, Barnett GH. Radiofrequency cingulotomy for intractable cancer pain using stereotaxis guided by magnetic resonance imaging. Neurosurgery. 1990;27(2):220–223. 2200976 10.1097/00006123-199008000-00008

[39] Pillay PK , Hassenbusch SJ. Bilateral MRI-guided stereotactic cingulotomy for intractable pain. Stereotact Funct Neurosurg. 1992;59(1–4):33–38. 1295044 10.1159/000098914

[40] Strauss I , Berger A, Ben Moshe S, et al Double anterior stereotactic cingulotomy for intractable oncological pain. Stereotact Funct Neurosurg. 2017;95(6):400–408. 29316566 10.1159/000484613

[41] Patel NV , Agarwal N, Mammis A, Danish SF. Frameless stereotactic magnetic resonance imaging-guided laser interstitial thermal therapy to perform bilateral anterior cingulotomy for intractable pain: Feasibility, technical aspects, and initial experience in 3 patients. Oper Neurosurg (Hagerstown). 2015;11(Suppl 2):17–25. 25584953 10.1227/NEU.0000000000000581

[42] Yen CP , Kung SS, Su YF, Lin WC, Howng SL, Kwan AL. Stereotactic bilateral anterior cingulotomy for intractable pain. J Clin Neurosci. 2005;12(8):886–890. 16326270 10.1016/j.jocn.2004.11.018

[43] Wang GC , Harnod T, Chiu TL, Chen KP. Effect of an anterior cingulotomy on pain, cognition, and sensory pathways. World Neurosurg. 2017;102:593–597. 28342924 10.1016/j.wneu.2017.03.053

[44] Tsai MD , Wang AJ, Wei CP, Tsai MC. 428 neuropathic pain following spinal cord trauma treated with cingulotomy: Report of two cases. Eur J Pain Suppl. 2010;4(S1):121–122.

[45] Pereira EA , Paranathala M, Hyam JA, Green AL, Aziz TZ. Anterior cingulotomy improves malignant mesothelioma pain and dyspnoea. Br J Neurosurg. 2014;28(4):471–474. 24199940 10.3109/02688697.2013.857006

[46] Hochberg U , Berger A, Atias M, Tellem R, Strauss I. Tailoring of neurosurgical ablative procedures in the management of refractory cancer pain. Reg Anesth Pain Med. 2020;45(9):696–701. 32699105 10.1136/rapm-2020-101566

[47] Faillace LA , Allen RP, McQueen JD, Northrup B. Cognitive deficits from bilateral cingulotomy for intractable pain in man. Dis Nerv Syst. 1971;32(3):171–175. 4929083

[48] Voris HC , Whisler WW. Results of stereotaxic surgery for intractable pain. Confin Neurol. 1975;37(1–3):86–96. 1093800 10.1159/000102718

[49] Brown MH . Limbic target surgery in the treatment of intractable pain with drug addiction. Springer; 1977:233–233.

[50] Foltz EL , White LE. Experimental cingulumotomy and modification of morphine withdrawal. J Neurosurg. 1957;14:655–670. 13476224 10.3171/jns.1957.14.6.0655

[51] Adams JL , Goble G, Amy J. Multidisciplinary approaches: Cingulotomy in an adult with refractory neuropathic cancer-related pain. J Palliat Med. 2023;26(9):1297–1301. 37192484 10.1089/jpm.2022.0444

[52] Foltz EL , White LE. The role of rostral cingulumotomy in “pain” relief. Int J Neurol. 1968;6(3–4):353–373. 5759640

[53] Farrell SM , Green A, Aziz T. The current state of deep brain stimulation for chronic pain and its context in other forms of neuromodulation. Brain Sci. 2018;8(8):158. 30127290 10.3390/brainsci8080158 PMC6119957

[54] Spooner J , Yu H, Kao C, Sillay K, Konrad P. Neuromodulation of the cingulum for neuropathic pain after spinal cord injury. Case report. J Neurosurg. 2007;107(1):169–172. 17639889 10.3171/JNS-07/07/0169

[55] Coenen VA , Schlaepfer TE, Allert N, Madler B. Diffusion tensor imaging and neuromodulation: DTI as key technology for deep brain stimulation. Int Rev Neurobiol. 2012;107:207–234. 23206684 10.1016/B978-0-12-404706-8.00011-5

[56] Boccard SG , Fernandes HM, Jbabdi S, et al Tractography study of deep brain stimulation of the anterior cingulate Cortex in chronic pain: Key to improve the targeting. World Neurosurg. 2016;86:361–370.e1-3. 26344354 10.1016/j.wneu.2015.08.065

[57] Mohseni HR , Smith PP, Parsons CE, et al MEG can map short and long-term changes in brain activity following deep brain stimulation for chronic pain. PLoS One. 2012;7(6):e37993. 22675503 10.1371/journal.pone.0037993 PMC3366994

[58] Ray N , Jenkinson N, Kringelbach M, et al Abnormal thalamocortical dynamics may be altered by deep brain stimulation: Using magnetoencephalography to study phantom limb pain. J Clin Neurosci. 2009;16(1):32–36. 19019684 10.1016/j.jocn.2008.03.004

[59] Kringelbach ML , Jenkinson N, Owen SL, Aziz TZ. Translational principles of deep brain stimulation. Nat Rev Neurosci. 2007;8(8):623–635. 17637800 10.1038/nrn2196

[60] Pereira E , Green A. Autonomic neurosurgery: From microvascular decompression to image guided stimulation. Biomed Imaging Interv J. 2007;3(1):e14. 21614256 10.2349/biij.3.1.e14 PMC3097652

[61] Boccard SG , Pereira EA, Moir L, et al Deep brain stimulation of the anterior cingulate cortex: Targeting the affective component of chronic pain. Neuroreport. 2014;25(2):83–88. 24100411 10.1097/WNR.0000000000000039

[62] McBenedict B , Petrus D, Pires MP, et al The role of the Insula in chronic pain and associated structural changes: An integrative review. Cureus. 2024;16(4):e58511. 38770492 10.7759/cureus.58511 PMC11103916

[63] Barrett LF , Simmons WK. Interoceptive predictions in the brain. Nat Rev Neurosci. 2015;16(7):419–429. 26016744 10.1038/nrn3950 PMC4731102

[64] Geisler M , Rizzoni E, Makris N, et al Microstructural alterations in medial forebrain bundle are associated with interindividual pain sensitivity. Hum Brain Mapp. 2021;42(4):1130–1137. 33170528 10.1002/hbm.25281 PMC7856635

[65] Galhardoni R , da Silva VA, García-Larrea L, et al Insular and anterior cingulate cortex deep stimulation for central neuropathic pain: Disassembling the percept of pain. Neurology. 2019;92(18):e2165–e2175. 30952795 10.1212/WNL.0000000000007396

[66] Levi V , Cordella R, D'Ammando A, et al Dorsal anterior cingulate cortex (ACC) deep brain stimulation (DBS): A promising surgical option for the treatment of refractory thalamic pain syndrome (TPS). Acta Neurochir (Wien). 2019;161(8):1579–1588. 31209628 10.1007/s00701-019-03975-5

[67] Boccard SG , Fitzgerald JJ, Pereira EA, et al Targeting the affective component of chronic pain: A case series of deep brain stimulation of the anterior cingulate cortex. Neurosurgery. 2014;74(6):628–635. 24739362 10.1227/NEU.0000000000000321

[68] Ballantine HT Jr , Bouckoms AJ, Thomas EK, Giriunas IE. Treatment of psychiatric illness by stereotactic cingulotomy. Biol Psychiatry. 1987;22(7):807–819. 3300791 10.1016/0006-3223(87)90080-1

[69] Zhang Q , Wang W, Wei X. Long-term efficacy of stereotactic bilateral anterior cingulotomy and bilateral anterior capsulotomy as a treatment for refractory obsessive-compulsive disorder. Stereotact Funct Neurosurg. 2013;91(4):258–261. 23652367 10.1159/000348275

[70] Kierońska-Siwak S , Sokal P, Jabłońska M, Rudaś M, Bylinka A. Structural connectivity reorganization based on DTI after cingulotomy in obsessive-compulsive disorder. Brain Sci. 2022;13(1):44. 36672026 10.3390/brainsci13010044 PMC9856478

[71] Plun-Favreau H , Lewis PA, Hardy J, Martins LM, Wood NW. Cancer and neurodegeneration: Between the devil and the deep blue sea. PLoS Genet. 2010;6(12):e1001257. 21203498 10.1371/journal.pgen.1001257 PMC3009676

[72] Steele JD , Christmas D, Eljamel MS, Matthews K. Anterior cingulotomy for major depression: Clinical outcome and relationship to lesion characteristics. Biol Psychiatry. 2008;63(7):670–677. 17916331 10.1016/j.biopsych.2007.07.019

[73] Peters J , Tisch S. Habituation after deep brain stimulation in tremor syndromes: Prevalence, risk factors and long-term outcomes. Front Neurol. 2021;12:696950. 34413826 10.3389/fneur.2021.696950 PMC8368435

[74] Fasano A , Helmich RC. Tremor habituation to deep brain stimulation: Underlying mechanisms and solutions. Mov Disord. 2019;34(12):1761–1773. 31433906 10.1002/mds.27821

[75] Hamani C , Lozano AM. Hardware-related complications of deep brain stimulation: A review of the published literature. Stereotact Funct Neurosurg. 2006;84(5–6):248–251. 17063047 10.1159/000096499

[76] Henderson JM , Heit G, Fisher RS. Recurrent seizures related to motor cortex stimulator programming. Neuromodulation. 2010;13(1):37–43. 21992763 10.1111/j.1525-1403.2009.00256.x

[77] Oderiz CC , von Ellenrieder N, Dubeau F, et al Association of cortical stimulation–induced seizure with surgical outcome in patients with focal drug-resistant epilepsy. JAMA Neurol. 2019;76(9):1070–1078. 31180505 10.1001/jamaneurol.2019.1464 PMC6563597

[78] Vitek JL . Mechanisms of deep brain stimulation: Excitation or inhibition. Mov Disord. 2002;17(Suppl 3):S69–S72. 11948757 10.1002/mds.10144

[79] Deng Z-D , Lisanby SH, Peterchev AV. Electric field depth–focality tradeoff in transcranial magnetic stimulation: Simulation comparison of 50 coil designs. Brain Stimul. 2013;6(1):1–13. 22483681 10.1016/j.brs.2012.02.005 PMC3568257

[80] Hallett M . Transcranial magnetic stimulation: A primer. Neuron. 2007;55(2):187–199. 17640522 10.1016/j.neuron.2007.06.026

[81] Barthas F , Sellmeijer J, Hugel S, Waltisperger E, Barrot M, Yalcin I. The anterior cingulate cortex is a critical hub for pain-induced depression. Biol Psychiatry. 2015;77(3):236–245. 25433903 10.1016/j.biopsych.2014.08.004

[82] Tzabazis A , Aparici CM, Rowbotham MC, Schneider MB, Etkin A, Yeomans DC. Shaped magnetic field pulses by multi-coil repetitive transcranial magnetic stimulation (rTMS) differentially modulate anterior cingulate cortex responses and pain in volunteers and fibromyalgia patients. Mol Pain. 2013;9:33. 23819466 10.1186/1744-8069-9-33 PMC3750766

[83] Quesada C , Fauchon C, Pommier B, et al Field recordings of transcranial magnetic stimulation in human brain postmortem models. Pain Rep. 2024;9(2):e1134. 38375090 10.1097/PR9.0000000000001134 PMC10876241

[84] Hayward G , Mehta MA, Harmer C, Spinks TJ, Grasby PM, Goodwin GM. Exploring the physiological effects of double-cone coil TMS over the medial frontal cortex on the anterior cingulate cortex: An H215O PET study. Eur J Neurosci. 2007;25(7):2224–2233. 17439499 10.1111/j.1460-9568.2007.05430.x

