# Supplementary material for: The cingulum: a central hotspot for the battle against chronic intractable pain?
Source: Brain Commun. 2024 Oct 16;6(5):fcae368. doi: 10.1093/braincomms/fcae368 (PMC11522883; doi:10.1093/braincomms/fcae368)
Supplement: fcae368_Supplementary_Data [file fcae368_supplementary_data.docx]

*Supplementary Table 1: Overview of studies on cingulotomy for neoplastic and non-neoplastic (chronic) pain; target location and technique*

| *Study* | *No. of lesions* | *Coronal plane, mm above ventrical roof* | *Sagittal plane; mm posterior to tip of frontal horn* | *Horizontal plan; mm from midline* | *Remarks targetING* | *Guidance* | | *Material* | *Temperature* | *Lesioning time* | *Lesion size* |
| --- | --- | --- | --- | --- | --- | --- | --- | --- | --- | --- | --- |
| *Foltz & White 1962^1^* | Unilateral (5 patients), bilateral (11 patients).  In first 6 patients 3 lesions were made anteriorly and posteriorly from the initial point. | 5.5 (5-6) | 17.5 (10-25) | 13 | To determine the optimal point for entrance of the electrode, a mid-line measurement was made from the nasion 9 cm posteriorly to a point just anterior to the coronal suture. Electrodes were inserted 1.5 cm laterally from this line, on each side. | Fractional pneumoencephalography or ventrigulography | | Electrocoagulation (Bovie) | N/A | 20-30s | N/A |
| *Ballantine et al 1967^2^* | Bilateral, 2 per side | 20 and 2^nd^ lesion 10 below first lesion. | 32.5 (25-40) | 7.5 (5-10) lateral to midline | N/A | Ventriculography | | Monopolar radiofrequency current | 2-8W | 15-30s (later 60s) | Lesion extent about 2cm |
| *Foltz & White 1968^3^* | Bilateral | 95-100 posterior to nasion | 25, in case of second lesion further posteriorly | 13 | N/A | Ventriculography | | Radiofrequency | 45-50C | N/A | Approximately 1.2cm in diameter |
| *Faillace et al 1971^4^* | Bilateral | N/A | 37.5 (35-40) | N/A | N/A | N/A | | Radiofrequency heat ablation | N/A | N/A | N/A |
| *Hurt & Ballantine 1974^5^* | Bilateral, 2 per side | 20 in height 1mm above roof | 30 (20-40) | N/A | N/A | Ventriculography | | Radiofrequency | 8W | 60-75s | Transverse diameter about 2cm with 60s |
| *Voris & Whisler 1975^6^* | Bilateral, 2 per side | 6 | 17.5 (10-25) (usually 13 and 17) | 12 | N/A | N/A | | N/A | N/A | N/A | N/A |
| *Hassenbusch et al 1990^7^* | Bilateral, 1 per side | N/A | N/A | N/A | N/A | MRI | | Radiofrequency electrode, 10mm exposed tip | 75C (1 patient 85C) | 60s (1 patient 90s) | 5x15mm cilinders |
| *Pillay & Hassenbusch 1992^8^* | Bilateral, 1 per side | N/A | N/A | N/A | N/A | MRI | | Radiofrequency electrode, 10mm exposed tip | 75C | 60s | 5x15mm cilinders |
| *Wong et al 1997^9^* | Bilateral, 1 per side | N/A | N/A | N/A | N/A | MRI | | Radiofrequency electrode, 10mm exposed tip | N/A | N/A | N/A |
| *Cohen et al 1999^10^* | Bilateral, 1 per side | N/A | N/A | slightly lateral to midline, 5mm diameter | neuroradiologic description of lesion location indicated that in all cases the lesion was almost entirely limited to the cingulate cortex, with only minimal involvement of the supplementary motor cortex | MRI | | Thermal probes | N/A | N/A | Mean total volume lesions: 855.4 ± 61.2mm3 (mean ± SD) |
| *Wilkinson et al 1999^11^* | Bilateral, 2 per side | 5, second lesion 10mm withdrawal | N/A | 5 | N/A | Ventriculography or CT | | Radiofrequency electrode, 10mm exposed tip, 12-gauge | 80C | 120s | N/A |
| *Yen et aL 2005^12^ & 2009^13^* | Bilateral, 1 or 2 per side | Second lesion 3mm above first | 24 | N/A | on target slice (24mm posterior to tip of frontal horn), coordinates of fiducials determined, center of each cingulate gyrus as target point | MRI | | Radiofrequency thermocoagulation with thermocoupled bipolar lesion electrode | 80C | 80s | N/A |
| *TSAI ET AL 2013^14^* | Bilateral | N/A | N/A | N/A | N/A | MRI | | N/A | N/A | N/A | N/A |
| *Pereira et al 2014^15^* | Bilateral, 2 per side | 1.5 (1-2), second lesion withdrawing 10mm | 20 | 10 | N/A | N/A | | Radiofrequency thermocoagulation electrode with 10mm uninsulated tip | 80C | 90s | Effective lesion: cylindrical, 20mm high, 8-10mm diameter |
| *Patel et al 2015^16^* | Bilateral, 2 per side | Mean 5.8, second 10-20mm above first lesion | Mean 20.8 | Mean 7.6 | N/A | MRI | | Laser induced thermal therapy | 10.0W (10-11) (median, range) | Mean 240s per cingulum | Mean lesion size 1440mm3 |
| *Strauss et al 2017^17^* | Bilateral, 1 lesion (10mm tip) or 2-3 adjacent lesions in each trajectory (2mm tip) | N/A | 20 (24 and 16) | 7.5 (7-8) | N/A | | MRI | Radiofrequency thermocoagulation electrode, 2mm diameter with 4mm exposed tip, later 10mm exposed tip | 80C | 80s | N/A |
| *Wang et al 2017^18^* | Bilateral, 1 per side (3 for reoperation) | N/A | 24 (reoperation: 5mm anterior and posterior to initial lesions) | N/A | N/A | | MRI | Radiofrequency bipolar electrode, 2mm diameter | 30W, 80C | 60s (reoperation 90s) | Right side median 7.35mm (4.3-8.5), left side median 7.05mm (4.6-9.2) |
| *Deng et al 2019^19^* | Bilateral, 5 per side | 1.5 (1-2), each lesion 4mm above the other to result in 'one' 20mm long lesion | 20, third lesion 5mm posterior to first | 6 (5-7), second lesion 5mm lateral to first, third lesion 2mm lateral to first | N/A | | MRI | Radiofrequency thermocoagulation electrode, 2.1mm diameter, 5mm naked tip | 80C | 60s | 5mm diameter, 20mm in length |
| *Hochberg et al 2020^20^* | Bilateral, 2 per side | N/A | 20 (24 and 16) | 7.5 (7-8) | N/A | | MRI | Radiofrequency thermocoagulation electrode, 10mm exposed tip | 80C | 60s | N/A |
| *jALON ET AL 2023^21^* | Bilateral, 1 lesion (10mm tip) or 2-3 adjacent lesions in each trajectory (2mm tip) | N/A | 20 (24 and 16) | 20 (24 and 16) | N/A | | MRI | Radiofrequency thermocoagulation electrode, 2mm diameter with 4mm exposed tip, later 10mm exposed tip | 80c | 80s | N/A |
| *Kollenburg et al 2024^22^* | Bilateral, 2 lesions on the left and 3 on the right side | N/A | 24 | 6 lateral to midline | N/A | | MRI | Radiofrequency thermocoagulation electrode, 5mm exposed tip | 75C | 60s | N/A |

N/A, not available.

| Study  *Supplementary Table 2: Overview on studies of cingulotomy for neoplastic and non-neoplastic (chronic) pain; patients, effect and complications* | Study design | No of patients | Age | Diagnosis | Follow-up time | DEFENITION Responders | Results | Complications/side-effects | Remarks |
| --- | --- | --- | --- | --- | --- | --- | --- | --- | --- |
| Foltz & White 1962^1^ | CS | 16 | N/A | **Neoplastic (6):** 3 face/neck, 2 back/hip, 1 shoulder and arm pain  **Non-neoplastic (10):** 2 face, 2 vaginal-perineal, 1 chest, 1 arm causalgia, 1 'emotional angina', 1 atypical causalgia thigh stump, 2 leg pain | 4d – 7y | Good and/or excellent results | **Neoplastic:** 1m 4/6, 5m 1/2 good-excellent pain relief  **Non-neoplastic:** 3m 6/10, 5m 6/9, 2y 4/6, 7y 1/1 good-excellent pain relief | Often mild elevation in temperature (100-101⁰F) in second 24h postop. Some mild confusion 1-2d, usually change in affect | 1 reoperation in non-neoplastic group (good outcome) |
| Ballantine et al 1967^2^ | CS | 69 | 15-83 | Incurable cancer (12), mental illness (57) | 3m-4y | N/A | N/A | ‘Confused state’ for 2-5d postop (2). Postoperative seizures (3 of total 69, including psychiatric patients, of which 2 with previous history) | N/A |
| Foltz & White 1968^3^ | CS | 35 | N/A | **Neoplastic (11):** 1 throat, 1 tongue, 5 face/neck, 2 back-hip metastases, 1 shoulder/arm, 1 phantom limb pain  **Non-neoplastic (24):** 1 headache, 1 coccydynia, 3 face, 2 vaginal-perineal, 1 chest, 1 'burning feet', 3 causalgia, 1 'emotional angina', 1 pancreatitis, 3 legs (paraparesis, paraplegia), 1 thalamic face, 2 hemi-body, 1 chest hyperalgesia, 3 lumbar arachnoiditis pain | 1 – 9y | Good and/or excellent results | **Neoplastic:** 9/11 good-excellent pain relief  **Non-neoplastic:** 18/24 good-excellent pain relief | Mild hemiparesis with full recovery (1), reappearance Horner’s syndrome for 4m (1), urinary incontinence gradually controlled (4), hypotension 2d (1), disorientation in time 3-5d (6), complete hemiplegia (1, hemiparesis pre-existent), ventriculomegaly (1), tonic clonic seizure (1), suicide (2, with preoperative tendency) | 3 reoperations (successful; more caudal lesions) |
| Faillace et al 1971^4^ | CS | 9 | 51 (42-66) (median, range) | **Neoplastic (7):** terminal cancer (unspecified)  **Non-neoplastic (2):** intractable back pain | 3d – >2y | N/A | **Neoplastic:** 3/7 pain relief  **Non-neoplastic:** 1/2 pain relief | Decrement in tapping test (4/4 tested), decrement in Porteus maze after 2m (2) | N/A |
| Hurt & Ballantine 1974^5^ | CS | 68 | 56 (22-85) (mean, range) | **Neoplastic (32):** 4 mouth, 2 pharynx, 3 larynx, 7 lung, 3 colon, 3 pancreas, 3 uterus, 2 bladder, 2 melanoma, 1 liposarcoma, 1 osteosarcoma, 1 undifferentiated neoplasm  **Non-neoplastic (36):** 1 atypical facial, 1 central, 2 phantom limb, 2 tabetic, 4 postherpetic truncal/facial, 2 paraplegic, 8 arachnoiditis, 6 unknown visceral aetiology, 10 unknown somatic aetiology pain | 4d – 9y | ≥40% reduction in pain intensity | **Neoplastic:** ≤3m 18/32 (40%-100% improvement), >3m 2/9 (70-90% improvement)  **Non-neoplastic:** ≤3m 16/36 (40%-100% improvement), >3m 16/36 (40%-100% improvement)  14/28 reported partial or complete resumption of activity | Commonly headache and fever 1w postop. Less common transient bladder or bowel incontinence and transient confusion. Guillain-Barré syndrome (1) | 7 reoperations (6 patients).  Many neoplastic patients were deceased over 3 months (most in moderate-complete pain relief group) |
| Voris & Whisler 1975^6^ | CS | 16 | N/A | **Neoplastic (5):** N/A  **Non-neoplastic (11):** N/A | 1m – 12y | N/A | **Neoplastic:** 1-12m 5/5 pain relief  **Non-neoplastic:** 1-12m 8/11, 1-3y 2/11, >3y 1/11 pain relief | Hemiparesis (1), prolonged stupor (2), intracranial haemorrhage (1) | N/A |
| bROWN et al 1975^23^ | CS | 43^1^ | N/A | Intractable pain syndromes (4 phantom-limb) | 1-20y | Good and/or excellent results^2^ | 39/43 | N/A | N/A |
| Hassenbusch et al 1990^7^ | CS | 4 | 56 (49-65) (mean, range) | Diffuse bone pain secondary to widespread metastases (breast, spindle cell, lung cancer, multiple myeloma) | 2w – 4m | ≥50% reduction in pain medication intake | Immediate: 4/4 pain relief. 3/4 complete pain relief until time of death (2-6w), 1/4 excellent pain relief at last follow up (4m) | Aphasia 5d (1) | N/A |
| Pillay & Hassenbusch 1992^8^ | CS | 10 | 52 (24-69) (mean, range) | **Neoplastic (8):** 1 spindle cell, 1 lung, 3 breast, 1 rectal carcinoma; 1 chordoma, 1 myeloma (all with bony metastases).  **Non-neoplastic (2):** 1 neurofibromatosis, 1 thalamic stroke | 6m – 1y | Good and/or excellent results | **Neoplastic:** 6m 4/8 excellent  **Non-neoplastic:** 1y 1/2 good pain relief. | N/A | N/A |
| Wong et al 1997^9^ | CS | 3 | 46 (38-57) (mean, range) | Head and neck carcinoma with bony metastases, melanoma with spinal and epidural disease, cholangiocarcinoma with bone, lung, liver, cervical, supraclavicular lymph node metastases | N/A | ≥50% reduction in pain medication intake | 2/3 | 1 patient small bilateral haemorrhages and associated oedema near cingulotomy sites resulting in deficits in frontal lobe executive function, verbal learning, verbal declarative memory. | 1 patient also received Ommaya reservoir for intraventricular administration of morphine in same procedure |
| Cohen et al 1999^10^ | CC | 12 | 40-58 (range) | Noncerebral traumatic injury | 1y | N/A | 1y 8/12 modest pain relief | Mutism days (3), a-/bradykinesia and psychomotor slowing for days (8), blunting of affect for days (6), lethargy (2), changes in emotional behaviour, personality or cognitive ability (6; reported by families 1 year postoperatively) | N/A |
| Wilkinson et al 1999^11^ | CS | 23 | 32-77 (range) | Majority: lumbar or sciatic pain related to 'failed back syndrome' or adhesive arachnoiditis.  Others: phantom leg pain, venous occlusive disease, ischemic bilateral leg pain, 'failed neck' pain, atypical facial pain | 1 – 15y | ≥30% reduction in pain score | 2-9.5y: 20/23 | Flattening of affect and lack of spontaneity (23), transient aphasia 48h (2), urinary incontinence (3), repetitive hand washing days (1), intraoperative seizures (2), postoperative seizures (2), delayed seizures (5) (of which 4 controlled with medication) | 5 reoperations (3 successful) |
| Yen et al 2005^12^ | CS | 22 | 58.3 (35-79) (mean, range) | **Neoplastic (15):** 4 lung, 2 breast, 2 hepatocellular, 2 mesothelioma, 1 thyroid, 1 oesophageal, 1 ureteral, 1 lymphoma, 1 unknown primary (majority pain from widespread bony metastases)  **Non-neoplastic (7):** 2 diabetic neuropathy, 2 failed back surgery syndrome, 2 spinal cord injury, 1 trigeminal neuralgia | 1w – 1y | ≥30% reduction in pain intensity^2^ | **Neoplastic:** 1w 12/15, 1m 10/15, 3m 7/12, 6m 5/10  **Non-neoplastic:** 1w 7/7, 1m 5/7, 3m 5/7, 6m 5/7, 1y 5/7 | Transient confusion days (2), mild upper gastrointestinal bleeding (2, subsided with medication), some attentional impairment (5) | N/A |
| Yen et al 2009^13^ | CS | 10 | 64.4 (40-72) (mean, range) | Terminally ill cancer patients with intractable pain | 3m | ≥30% reduction in pain intensity^2^ | 1w 6/10, 1m 5/10, 3m 6/10 | Significant impairment of focused attention after 1w, improved at 1m.  Inappropriate, uninhibited speech (2, 2d), transient confusion (1) | Patients with somatic pain responded better than visceral pain |
| Tsai et al 2013^14^ | CR | 2 | N/A | Neuropathic pain secondary to cervical and thoracic spine trauma | N/A | N/A | 2/2 reported pain relief | Transient exacerbation of pain before gradual improvement (1) | N/A |
| Pereira et al 2014^15^ | CR | 1 | 67 | Malignant mesothelioma (chest pain) | 1-4m | ≥30% reduction in pain intensity | 1m: 1/1  4m: 1/1 | 24h mild confusion, incontinence of urine. At 1m numbness (not to light touch or pinprick) of chest wall | N/A |
| Patel et al 2015^16^ | CS | 3 | 45 (38-51) (mean, range) | Liposarcoma thigh with retroperitoneal and deep muscle metastases (back pain); breast cancer with liver, lung, brain metastases (pain left side chest); colorectal cancer with liver and lung metastases (abdominal pain) | 2w – 4m | ≥50% reduction in pain intensity | 2w 3/3, 6w 1/2, 4m 1/1 | N/A | 1 reoperation (successful; 20mm anterior to initial lesion, mean lesion size 2.73cm3 (vs 1.44)) |
| Strauss et al 2017^17^ | CS | 13 | 54 (31-78) (mean, range) | Oncological disease with diffuse pain syndromes: 3 abdomen/bone, 4 pelvis/lower limb, 4 spine/hip, 1 left arm/leg, 1 left hemibody | 1-3m | ≥50% reduction in pain intensity | Immediate 13/13^3^, 1m 8/11,  3m 5/7 | Transient confusion or mild apathy (4, 1w – 1m), transient urinary incontinence (2) | 1 reoperation (successful) |
| Wang et al 2017^18^ | CHR | 24 | 42 (24-75) (median, range) | 9 traumatic-related peripheral neuropathy or spinal cord injury, 6 spondylosis, 5 stroke-induced central neuropathic pain, 3 primary headache, 1 fibromyalgia | 51.5m (6-77) (median, range) | N/A | Initial median VAS 8, 1m 3, 3-6m 4, last follow-up 5 | Decline in language, executive function, or abstract thinking (4), decline in concentration and recent memory (1), decline in visuoconstructive skills (1). However, no significant differences in questionnaires pre- and postop. | No difference in patients with/without psychiatric disorders.  6 reoperations (no significant pain relief) |
| Deng et al 2019^19^ | CR | 2 | 56, 63 | Lumbar spinal stenosis (pain bilateral lower extremities and perineal region), diffuse pain after partial spinal cord injury | 18 – 60m | ≥50% reduction in pain intensity | 18m 2/2, 60m 1/1 | Confusion, headache, disorientation, fatigue (2, 1-2w) | Both patients also suffered from severe depression which improved |
| Hochberg et al 2020^20^ | CS | 23 | 56 (31-78) (mean, range) | Diffuse pain syndromes (19) or localized pain (4) due to widespread metastases: 5 lung, 5 colorectal, 3 sarcoma, 2 lymphoma, 2 cervical, 1 prostate, 1 breast, 6 others | 3m | ≥30% reduction in pain intensity | Immediate 20/22, 1m 16/19, 3m 7/12^3^ | Transient confusion or apathy (9), intracranial haemorrhage (1) | 4 patients with localized pain who underwent cingulotomy had unsatisfactory long-term results |
| Jalon ET AL 2023^21^ | CS | 12 | 56.67±12.03 | Patients with metastatic cancer | 1m | ≥50% reduction in pain intensity | Immediate 9/12, 1m 9/12 | N/A | N/A |
| Adams et al 2023^24^ | CR | 1 | 42 | Left lower extremity pain due to osteosarcoma | 1d-3d | ≥50% reduction in pain intensity | 1d 1/1, 3d 1/1 | N/A | A sudden death of the patient, caused by an aspiration event in the setting of small bowel obstruction secondary to malignant stricture, constrained the measurements of pain intensity at longer-follow up moments |
| kollenburg et al 2024^22^ | CR | 1 | 60 | Central post stroke pain | 3y | ≥50% reduction in pain intensity | 18w 1/1, 26w 1/1, 3y 1/1 | Progression of aphasia with full recovery after a few weeks (1/1) | N/A |

CS, case series; CC, case control; CR, case report; CHR, chart review; N/A, not available; d, days; w, weeks; m, months; y, years; h, hours; VAS, visual analogue scale.

^1^Although cingulotomy was performed in all subjects of the study, nine patients received additional lesions in the amygdaloid nucleus and two patients required innominate targets.

^2^ For this study only categories of <25%, 25-75% and/or >75% improvements in pain intensity were provided, hence their 30% response rate might also include some patients with 25-30% improvements.

^3^ For these/this value(s), the percentage pain relief was not mentioned for the individual patients.

| Study | Study design | No. of patients | Age | | Diagnosis | Follow up time | | Target | | Technique | Stimulation parameters | defenition responders | Results | Complications/side effects | Remarks | |
| --- | --- | --- | --- | --- | --- | --- | --- | --- | --- | --- | --- | --- | --- | --- | --- | --- |
| Spooner et al 2007^25^ | CR | 1 | 40 | | Spinal cord injury C4 | 2-4m | | Bilateral, 20mm posterior to anterior margin of the lateral ventricles in the midsection of the gyrus | | 4 lead contacts (Medtronic #3387), stereotactic | 130Hz | ≥30% reduction in pain intensity and/or pain medication intake | 2m: 1/1 (VAS 50% decrease and pain medication use 11% decrease)  4m: 1/1 (VAS 63% decrease and pain medication use 56% decrease)^1^ | None | Simultaneously implanted with right PVG DBS, but ACC stimulation had better effect compared to PVG or medication alone | |
| Boccard et al 2014^26^ | CR | 1 | 49 | | Traumatic brachial plexus injury | 2y | | Bilateral, 20mm posterior to anterior tip of frontal horns of lateral ventricles, contacts in cingulum bundle, deepest contact in corpus callosum | | Pre-operative MRI, stereotactic, Medtronic #3387 depth lead (1.27mm diameter, 4 lead contacts of 1.5mm and 1.5mm apart) | 2.5V, 130Hz, 330us; later on 4V, 130Hz, 450us | ≥30% reduction in pain intensity | 1y: 1/1 (VAS 40% decrease)  2y: 1/1 ( VAS 55% decrease, MPQ 42% decrease, SF-36 2.1% increase, EQ-5D unchanged) | 2y: Stroop Task improvement, increase in apathy and executive dysfunction | At 3V extreme tenderness to soles of feet.  No other differences in neurocognitive functioning (except for improvement of Stroop Task).  Deepest contacts active | |
| Boccard et al 2014^27^ | CS | 16 | 48.7 (33-63) (mean, range) | | 6 FBSS, 4 PSP, 3 brachial plexus injury, 1 cervical SCI, 1 head injury, 1 unknown chest pain | 13.2m (1-36m) (mean, range) | | Bilateral, 20mm posterior to anterior tip of frontal horns of lateral ventricles, contacts mostly in cingulum bundle, deepest contact in corpus callosum | | Pre-operative MRI, stereotactic, Medtronic #3387 depth lead (1.27mm diameter, 4 lead contacts of 1.5mm and 1.5mm apart) | Mean & range: 5V (4-6.9), 128.6Hz (120-130), 450us across all 4 contacts | ≥30% reduction in pain intensity | 5/11 (VAS 24.5% decrease, SF-36 7.3% increased (however physical functioning increased significantly), EQ-5D 20.3% improvement, MPQ 16.0% improvement)) | Infection (1, removed), increased pain post operatively (3/11) | 1 patient not implanted  1 removed due to inability to comply with treatment  Several patients described feeling as if their pain was separate from them physically and did not think about it anymore | |
| Boccard et al 2017^28^ | CS | 24 | 49.1 (21-72) (mean, range) | | 9 PSP, 6 FBSS, 3 brachial plexus injury, 2 SCI, 2 road traffic accident, 1 head injury, 1 unknown chest pain | 39.2m (24-65m) (mean, range) | | Bilateral (21/24 patients), only left (2), only right (1), 20mm posterior to anterior tip of frontal horns of lateral ventricles, contacts mostly in cingulum bundle, deepest contact in corpus callosum | | Pre-operative MRI, stereotactic, Medtronic #3387 depth lead (1.27mm diameter, 4 lead contacts of 1.5mm and 1.5mm apart) | Range: 4-6.5V, 130Hz, 450us | ≥30% reduction in pain intensity | 6m 7/11, 12m 5/8, 24m 0/4, 36m 2/8  2 patients not implanted after trial period (no pain relief)  6m: NRS 45% decrease, MPQ 36% decrease, EQ-5D 21% decrease, SF-36 physical functioning 54.2% improvement  10/12 (83.3%) implanted patients reported substantial pain relief  Generalized loss of efficacy at longer follow-up  Mild improvement in reasoning (1/8), verbal memory (2/8), Stroop (2/8), FAS (1/8), semantic fluency (2/8) and significant improvement in visual memory (1/8), Stroop (1/8), psychomotor speed (1/8) and mood (1/8)^2^ | Infection (5, removed), broken leads (2, 1 removal), stimulation induced seizures (2), de novo stimulation-induced epilepsy (2, even continuing after cessation stimulation), 2 other seizures (1 alcohol withdrawal & in 1 events ceased after explantation due to infection)  Mild decline in semantic fluency (3/8 tested), psychomotor speed (2/8), FAS (1/8) and significant decline in Stroop (1/8) | Follow-up of 10 patients unavailable due to removal: infection (4), diminished pain relief (3), further strokes making it ineffective (1), refusion to participate in study (2).  FBSS patients was significantly better than PSP patients for BP, vitality and mental health on SF-36.  Several patients described feeling as if their pain was separate from them physically and did not think about it anymore | |
| Levi et al 2019^29^ | CS | 5 | | 56.2 (47-66) (mean, range) | Thalamic ischemic lesions causing hemi-body and hemi-facial pain | | 18m | | Bilateral, 20mm posterior to the anterior tip of the frontal horns of the lateral ventricles, contacts mostly in cingulum bundle, deepest contact in corpus callosum | Pre-operative MRI, stereotactic, 4 lead contacts | Mean & range: 4.5V (4-5.5), 130Hz, 450us on all 4 leads | ≥30% reduction in pain intensity | 6m: 2/5 (NRS 38% decrease, MPQ 16.2% decrease, SF-36 24% increase, EQ-5D pain 23% decrease)  18m: 2/5 (NRS 35% decrease, MPQ 17.1% decrease, SF-36 4.1% increase, EQ-5D pain 23% decrease) | None | | Three-dimensional lead reconstruction showed correct placement in anterior cingulate cortex for all 10 leads |

*Supplementary Table 3: Overview of studies on DBS-ACC for chronic pain; patients, effect and complications*

CR, case report; CS, case series; N/A, not available; *d, days; w, weeks; m, months; y, years; h, hours; NRS, numeric rating scale; VAS, visual analogue scale.*

^1^ As the preoperative VAS score was not mentioned, percentages were calculated based on the average VAS scores measured at 2 and 4 m postoperatively when the stimulator was turned off.

^2^ Due to the lack of individual patient data, these response rates were calculated based on the box-plots provided in the article.

References

1. Foltz EL, White LE, Jr. Pain "relief" by frontal cingulumotomy. *J Neurosurg*. Feb 1962;19:89-100. doi:10.3171/jns.1962.19.2.0089
2. Ballantine HT, Cassidy WL, Flanagan NB, Marino R. Stereotaxic anterior cingulotomy for neuropsychiatric illness and intractable pain. *Journal of neurosurgery*. 1967;26(5):488-495.
3. Foltz EL. Current status and use of rostral cingulumotomy. *South Med J*. Sep 1968;61(9):899-908.
4. Faillace LA, Allen RP, McQueen JD, Northrup B. Cognitive deficits from bilateral cingulotomy for intractable pain in man. *Diseases of the nervous system*. Mar 1971;32(3):171-5.
5. Hurt RW, Ballantine HT, Jr. Stereotactic anterior cingulate lesions for persistent pain: a report on 68 cases. *Clin Neurosurg*. 1974;21:334-51.
6. Voris HC, Whisler WW. Results of stereotaxic surgery for intractable pain. *Confin Neurol*. 1975;37(1-3):86-96. doi:10.1159/000102718
7. Hassenbusch SJ, Pillay PK, Barnett GH. Radiofrequency cingulotomy for intractable cancer pain using stereotaxis guided by magnetic resonance imaging. *Neurosurgery*. Aug 1990;27(2):220-3. doi:10.1097/00006123-199008000-00008
8. Pillay PK, Hassenbusch SJ. Bilateral MRI-guided stereotactic cingulotomy for intractable pain. *Stereotact Funct Neurosurg*. 1992;59(1-4):33-8. doi:10.1159/000098914
9. Wong ET, Gunes S, Gaughan E, et al. Palliation of intractable cancer pain by MRI-guided cingulotomy. *Clin J Pain*. Sep 1997;13(3):260-3.
10. Cohen RA, Kaplan RF, Moser DJ, Jenkins MA, Wilkinson H. Impairments of attention after cingulotomy. *Neurology*. Sep 11 1999;53(4):819-24. doi:10.1212/wnl.53.4.819
11. Wilkinson HA, Davidson KM, Davidson RI. Bilateral anterior cingulotomy for chronic noncancer pain. *Neurosurgery*. Nov 1999;45(5):1129-34; discussion 1134-6. doi:10.1097/00006123-199911000-00023
12. Yen CP, Kung SS, Su YF, Lin WC, Howng SL, Kwan AL. Stereotactic bilateral anterior cingulotomy for intractable pain. *J Clin Neurosci*. Nov 2005;12(8):886-90. doi:10.1016/j.jocn.2004.11.018
13. Yen CP, Kuan CY, Sheehan J, et al. Impact of bilateral anterior cingulotomy on neurocognitive function in patients with intractable pain. *J Clin Neurosci*. Feb 2009;16(2):214-9. doi:10.1016/j.jocn.2008.04.008
14. Tsai MD, Wang AJ, Wei CP, Tsai MC. 428 NEUROPATHIC PAIN FOLLOWING SPINAL CORD TRAUMA TREATED WITH CINGULOTOMY: REPORT OF TWO CASES. *European Journal of Pain Supplements*. 2010;4(S1):121-122.
15. Pereira EA, Paranathala M, Hyam JA, Green AL, Aziz TZ. Anterior cingulotomy improves malignant mesothelioma pain and dyspnoea. *Br J Neurosurg*. Aug 2014;28(4):471-4. doi:10.3109/02688697.2013.857006
16. Patel NV, Agarwal N, Mammis A, Danish SF. Frameless stereotactic magnetic resonance imaging-guided laser interstitial thermal therapy to perform bilateral anterior cingulotomy for intractable pain: feasibility, technical aspects, and initial experience in 3 patients. *Operative neurosurgery (Hagerstown, Md)*. Mar 2015;11 Suppl 2:17-25; discussion 25. doi:10.1227/neu.0000000000000581
17. Strauss I, Berger A, Ben Moshe S, et al. Double Anterior Stereotactic Cingulotomy for Intractable Oncological Pain. *Stereotact Funct Neurosurg*. 2017;95(6):400-408. doi:10.1159/000484613
18. Wang GC, Harnod T, Chiu TL, Chen KP. Effect of an Anterior Cingulotomy on Pain, Cognition, and Sensory Pathways. *World Neurosurg*. Jun 2017;102:593-597. doi:10.1016/j.wneu.2017.03.053
19. Deng Z, Pan Y, Li D, et al. Effect of Bilateral Anterior Cingulotomy on Chronic Neuropathic Pain with Severe Depression. *World Neurosurg*. Jan 2019;121:196-200. doi:10.1016/j.wneu.2018.10.008
20. Hochberg U, Berger A, Atias M, Tellem R, Strauss I. Tailoring of neurosurgical ablative procedures in the management of refractory cancer pain. *Reg Anesth Pain Med*. Sep 2020;45(9):696-701. doi:10.1136/rapm-2020-101566
21. Jalon I, Berger A, Shofty B, et al. Lesions to both somatic and affective pain pathways lead to decreased salience network connectivity. *Brain*. 2022;146(5):2153-2162. doi:10.1093/brain/awac403
22. Kollenburg L, Kurt E, Arnts H, Vinke S. Cingulotomy: the last man standing in the battle against medically refractory poststroke pain. *PAIN Reports*. 2024;9(2):e1149. doi:10.1097/pr9.0000000000001149
23. Brown MH. Limbic target surgery in the treatment of intractable pain with drug addiction. Springer; 1977:233-233.
24. Jessica L. Adams GG, and Amy Johnson. Multidisciplinary Approaches: Cingulotomy in an Adult With Refractory Neuropathic Cancer-Related Pain. *Journal of Palliative Medicine*. 2023;26(9):1297-1301. doi:10.1089/jpm.2022.0444
25. Spooner J, Yu H, Kao C, Sillay K, Konrad P. Neuromodulation of the cingulum for neuropathic pain after spinal cord injury. Case report. *Journal of neurosurgery*. Jul 2007;107(1):169-72. doi:10.3171/JNS-07/07/0169
26. Boccard SG, Pereira EA, Moir L, et al. Deep brain stimulation of the anterior cingulate cortex: targeting the affective component of chronic pain. *Neuroreport*. Jan 22 2014;25(2):83-8. doi:10.1097/WNR.0000000000000039
27. Boccard SG, Fitzgerald JJ, Pereira EA, et al. Targeting the affective component of chronic pain: a case series of deep brain stimulation of the anterior cingulate cortex. *Neurosurgery*. Jun 2014;74(6):628-35; discussion 635-7. doi:10.1227/NEU.0000000000000321
28. Boccard SGJ, Prangnell SJ, Pycroft L, et al. Long-Term Results of Deep Brain Stimulation of the Anterior Cingulate Cortex for Neuropathic Pain. *World Neurosurg*. Oct 2017;106:625-637. doi:10.1016/j.wneu.2017.06.173
29. Levi V, Cordella R, D'Ammando A, et al. Dorsal anterior cingulate cortex (ACC) deep brain stimulation (DBS): a promising surgical option for the treatment of refractory thalamic pain syndrome (TPS). *Acta Neurochir (Wien)*. Jun 17 2019;doi:10.1007/s00701-019-03975-5
